# Supplementary material for: Selection Effects and COVID-19 Mortality Risk after Pfizer vs. Moderna Vaccination: Evidence from Linked Mortality and Vaccination Records
Source: Vaccines (Basel). 2023 May 11;11(5):971. doi: 10.3390/vaccines11050971 (PMC10221876; doi:10.3390/vaccines11050971)

**Online Appendix for**

# Selection Effects and COVID-19 Mortality Risk after Pfizer vs. Moderna Vaccination: Evidence from Linked Mortality and Vaccination Records

Vladimir Atanasov
William & Mary, Mason School of Business

Natalia Barreto
Northwestern University

Jeff Whittle
Medical College of Wisconsin

John Meurer
Medical College of Wisconsin

Benjamin W. Weston
Medical College of Wisconsin

Qian (Eric) Luo
George Washington University

Andy Ye Yuan *
Northwestern University, Pritzker School of Law

Lorenzo Franchi
Northwestern University

Ruohao Zhang
Northwestern University, Kellogg School of Management

Bernard Black
Northwestern University, Pritzker School of Law and Kellogg School of Management

Draft May 2023

Northwestern University, Pritzker School of Law
Law and Economics Research Paper No. 22-17

Northwestern University, Institute for Policy Research
Working Paper 23-02

*This Appendix can be downloaded from:*
http://ssrn.com/abstract=4321773

*The paper can be downloaded without charge from SSRN at:*
http://ssrn.com/abstract=4321768

* Corresponding author. andyyuan@law.northwestern.edu. Author order follows medical journal conventions, with principal credit to first, second, and last author.

# Online Appendix for

# Selection Effects and COVID-19 Mortality Risk after Pfizer vs. Moderna Vaccination: Evidence from Linked Mortality and Vaccination Records

Vladimir Atanasov[b], Paula Natalia Barreto Parra[e], Jeff Whittle[c], John Meurer[c], Benjamin Weston[c], Qian (Eric) Luo[d], Andy Ye Yuan[a], Lorenzo Franchi[a], Ruohao Zhang[f], and Bernard Black[a]

[a]Northwestern University; [b]William & Mary; [c]Medical College of Wisconsin; [d]George Washington University; [e]University of Illinois, Urbana-Champaign; [f] Pennsylvania State University

**Abstract:** This Appendix provides additional data source and methods details and additional results for Atanasov et. al, Relative Effectiveness of COVID-19 Vaccines from Moderna and Pfizer: Evidence from Linked Mortality and Vaccination Records (working paper 2023).

The underlying paper is available at http://ssrn.com/abstract=4321773

## Contents

Additional Literature Discussion .......... 3
Data Sources, Data Linkage, and Match Accuracy .......... 4
Extended Methods: Time Period, Variable Definitions. and Other Details .......... 5
Identifying COVID-19 Decedents from Cause of Death Fields .......... 7
Virus Variants and Periods of Dominance .......... 8
Measuring Population by Month and Number of Vaccine Doses .......... 8
Time Periods and Booster Rollout .......... 9
Details on Younger, Fully Vaccinated COVID-19 Decedents .......... 9
Vaccination Counts and Rates .......... 9
Confidence Intervals for Text Table 2 .......... 10
Defining the Immune-Compromised .......... 10
Lag Period Between Vaccine Administration and Effectiveness Against Death .......... 10
Results by Gender .......... 11
Results for Whites versus Other Racial/Ethnic Groups .......... 11
Non-Covid Natural Mortality in 2019 as Predictor of COVID-19 Mortality in Pre-Vaccine Period .......... 11
Evidence on Selection Effects from Non-Covid-NMRs .......... 12
Additional Evidence on Selection Effects: CEMP vs. Covid-PFR as Outcome .......... 12
Reporting Accuracy for COVID-19 Deaths and Excess Non-COVID-19 Natural Mortality .......... 12
Additional References for Online Appendix (not cited in the text) .......... 13
Table S1. Comparison of COVID-19 deaths per text analysis, to ICD-10 codes .......... 14
Table S2. Summary Statistics on Vaccination Status and Vaccine Type .......... 15
Table S3. Confidence Intervals for Pfizer/Moderna Ratio in Text Table 2 .......... 16
Table S4. Multivariate Logit Model with Additional Covariates .......... 17
Table S5. RMRs and Pfizer/Moderna Ratio by Age Group and Time Period, Including Immune-Compromised .......... 18
Table S6. RMRs and Pfizer/Moderna Ratio by Age Group and Time Period .......... 19
Table S7. RMRs and Pfizer/Moderna Ratio by Gender .......... 20
Table S8. CEMP and RMR by Non-Hispanic White vs. Other Race/Ethnicity .......... 22
Table S9. Non-Covid Natural Mortality Rate (Non-Covid-NMR) by Vaccine Type, Age Group, and Time Period .......... 24
Table S10. Covid-19 PFR by Age Group and Time Period .......... 25
Figure S1. Sample selection flowchart .......... 26
Figure S2. Actual versus Predicted Non-COVID Natural Mortality in Wisconsin .......... 27
Figure S3. Actual versus Predicted Non-COVID-19 Natural Mortality Rates: Indiana .......... 28
Figure S4. Vaccination Rates for Adults by Age Group in Milwaukee County .......... 29
Figure S5. Correlation between 2019 Natural Mortality and 2020 COVID-19 Mortality .......... 30

## Additional Literature Discussion

As noted in the text, we found only one U.S. study that relied on linked mortality and vaccination records and reported VE or RMR (Robles-Fontan et. al, 2022). This study did not

control for selection effects. We also found a small study of Los Angeles County for May-July 2021, which had only 24 deaths among vaccinated persons and does not report RMR against death (Griffin et. al, 2021). Lin et al. (2022) have vaccination data for the entire population of North Carolina, but no individual health data and only a partial link to mortality records.

As noted in the text, we found only one study of veterans during 3Q-2021 Delta period, which found two-dose RMRs similar to those we report, for veterans seen at the VA (Cohn et. al, 2021).[1] This study controlled for age, sex, race, ethnicity, and comorbidities (Charlson comorbidity score, overweight, type II diabetes, chronic obstructive pulmonary disease, bronchitis, acute respiratory failure, and chronic lung disease). These controls should reduce selection bias, and may on the whole, provide an adjustment for selection effects comparable to our use of CEMP, which controls for natural non-COVID mortality risk. This study found similar VE against mortality for Pfizer and Moderna (81.5% for Moderna, and 84.3% for Pfizer under age 65; 75.5% for Moderna and 70.1% for Pfizer for ages 65+).

Two additional VA studies (Dickerman et. al, 2022a, 2022b) study only Pfizer vs. Moderna (not vaccinated vs. unvaccinated) using a matching approach based on VA health records. They find a statistically insignificant two-dose Moderna advantage in reducing mortality over a 24-week period in 2021; and an insignificant three-dose Moderna advantage over limited time periods in late 2021 and early 2022.

**Data Sources, Data Linkage, and Match Accuracy**

We obtain individual, de-identified mortality records for Milwaukee County for 2017 through June 2022 from the Wisconsin Department of Vital Statistics, based on the underlying death certificates. We also obtain individual, de-identified COVID-19 vaccination records from the Wisconsin Immunization Registry for January 2021 through June 2022. The Wisconsin Immunization Registry (WIR) is administered by the Wisconsin Department of Health Services and is an all ages, confidential, computerized repository of individual immunization records that integrates information public and private health care providers as well as birth and death records, and has been in operation since 1999. The mortality records include 5-digit zip code of residence, age at death, gender, race/ethnicity, education, income, marital status, veteran status, manner of death, and text fields for primary cause of death, conditions contributing to death, and other significant conditions. These data sets are drawn from statewide data, so include all within-state deaths and immunizations of Milwaukee County residents. We use text analysis to identify deaths due to COVID-19; this approach counts more COVID-19 deaths than relying on ICD-10 cause-of-death codes, prepared by the National Center for Health Statistics (NCHS) based on the text fields. We treat vaccine doses as effective against mortality beginning 30 days after receipt, and exclude immune-compromised decedents. The two sets of records include age in years, gender, residence zip code, and linking "tokens" based on last name, first name, gender, and exact date of birth. In a small number of cases where first letter of first name was available but full first name was missing in one or both datasets, we accepted a match on last name, first initial of first name, gender, and date of birth.

Out of an estimated adult population of 722,000, of which 536,000 (74%) have vaccination records and are not immunocompromised (Table App-2), this match resulted in 1 apparent mismatch; a person who died of natural causes during our sample period, but was recorded as

[1] Cohn et. al (2022).

being vaccinated after death (although a correct match but an incorrect vaccination date is also possible).

For 955 people with vaccination records (0.18% of vaccinated persons), gender is reported as unknown in the vaccination records. The matching algorithm we use, from Datavant, treats missing gender as equivalent to male. Thus, if gender is male, the algorithm will match these people successfully to mortality records (which have complete data on gender), but if gender is female, no match will be found. Overall, we have 9,652 female deaths in our sample from natural causes (933 from COVID-19 and 8,719 from other natural causes). With an 0.18% missingness rate (assuming missingness is similar for men and women), we will fail to match approximately 2 female COVID-19 deaths and 16 other natural deaths to vaccination records due to this issue. We have no reason to believe that these mismatches will lead to bias in CEMP values.

We define natural deaths as those with natural, pending, or undetermined manner of death; of these, the vast majority (98.7%) have manner of death = natural. We exclude the remaining manner-of-death categories (accident, homicide, and suicide) (14.4% of all deaths).

To measure the Non-Covid natural mortality rate, we use population estimates for 2020 from the American Community Survey.

Figure App-1 provides a sample selection flowchart. The final sample is 8,250 natural deaths in Milwaukee County from April 1, 2021 through June 30, 2022, of non-immune-compromised persons aged 18+, of whom 729 died of COVID-19.

**Extended Methods: Time Period, Variable Definitions. and Other Details**

COVID-19 vaccines because available in the U.S. in early 2021, but initially with limited supply, which was generally reserved to healthcare workers, the elderly, other persons at high risk for severe COVID-19, and other persons in high-exposure occupations. Availability expanded greatly in April 2021 and vaccines because available to all who wanted them by May 2021. We study mortality beginning April 1, 2021. Given the roughly one-month minimum period between first and second doses for both vaccines (3 weeks for Pfizer, 4 weeks for Moderna), and the minimum 30-day period we impose from most recent dose to death in order to treat a death as post-vaccination, it was not feasible to start the sample period much earlier than this.

*CEMP, VE, and RMR*

We study the mRNA vaccines from Pfizer and Moderna, both of which use two initial doses; we exclude J&J vaccinees. We define the COVID-19 Excess Mortality Percentage (CEMP), by number of doses $v$, and vaccine type, indexed by $i$ ($i$ = Pfizer or Moderna), as the percentage increase in natural deaths due to COVID-19:

$$CEMP_{vi} = 100 \times \frac{COVID\text{-}19\ deaths_{vi}}{(Natural\ deaths - COVID19\ deaths)_{vi}} \quad (1)$$

We define VE and relative mortality risk after vaccination (RMR) by vaccine type, number of doses $v$, and time period $t$ as:

$$VE_{ivt} = \frac{(CEMP_{unvax,t} - CEMP_{vax,ivt})}{CEMP_{unvax,t}} \quad ; \quad RMR_{ivt} = 1 - VE_{ivt} = \frac{CEMP_{vax,ivt}}{CEMP_{unvax,t}} \quad (2)$$

We also compute the Pfizer/Moderna mortality risk ratio (P/M ratio) as:

$$\frac{P}{M} ratio_{vt} = \frac{CEMP_{Pfizer,vt}}{CEMP_{Moderna,vt}} = \frac{RMR_{Pfizer,vt}}{RMR_{Moderna,vt}} \quad (3)$$

We study two-dose vaccinees in each time period, and three-dose vaccinees for the periods when a booster dose was available: fourth quarter of 2021 ("4Q-2021") and the Omicron period (1H-2022). By using non-COVID natural deaths in the CEMP denominator, we treat the non-COVID natural mortality rate as a proxy for the overall health of a given group, which can control for selection effects in vaccination patterns, including who gets vaccinated, when, with which vaccine, and with how many doses. Because the sample is decedents, these estimates are, in effect, mortality-weighted; thus results for a broad age group give primary weight to older persons within that group.

CEMP represents the odds, for a population of interest, of dying from COVID-19 versus other natural causes.

$$\frac{\sum COV_i^{mort} = 1}{\sum COV_i^{mort} = 0}$$

Here $COV_i^{mort}$ equals 1 for COVID-19 decedents, 0 for decedents from other natural causes.

We define the RMR of one group *I* relative to another group *J* as the ratio of the CEMPs of the groups:

$$RMR_{I\ vs.J} = \frac{\sum COV_i^{mort} = 1}{\sum COV_i^{mort} = 0} \Big/ \frac{\sum COV_j^{mort} = 1}{\sum COV_j^{mort} = 0}$$

The ratio of CEMPs or RMRs for two different groups, such as two-dose Pfizer versus two-dose Moderna vaccinees, is an odds ratio, also obtainable directly from logistic regression. We both compute RMRs directly and conduct multivariate logistic regression analysis of how vaccine type affects RMR. The regression predictors are vaccine type; days since most-recent dose (minus 14 days); age, age$^2$, zip-code-level socio-economic status (zip-SES), measured using the Graham Social Deprivation Index,[5] gender, race/ethnicity, education level, marital status, and military veteran status. The regression model is [Stata: logistic]:

$$Prob(COV_i^{mort} = 1) = \boldsymbol{f}(\boldsymbol{VS_i}, \boldsymbol{X_i})$$

Because we have limited vaccinee deaths, especially for three-dose vaccinees and younger persons, we need to limit covariates in the logistic regressions to preserve degrees of freedom. We therefore measure race/ethnicity as non-Hispanic White ("White") versus other, and education as high-school or less versus some college or more.

To assess differences in underlying health between two groups, proxied by their mortality rate from other natural causes, we need to estimate population. We use population estimates for 2020 from the American Community Survey. We measure the number of people receiving 1, 2, or 3 vaccine doses; exclude those receiving J&J or heterologous vaccine types, and assume the remaining population is unvaccinated. We define non-Covid-NMR for a population which received *v* doses of vaccine type *i*, in time period *t*, as:

$$NonCovidNMR_{ivt} = \frac{(natural\ non\text{-}COVID\text{-}19\ deaths)_{ivt}}{Population_{ivt}} \quad (4)$$

Days since most recent dose is measured using vaccination date = actual data + 14 days, to allow for full vaccine effectiveness.

**Identifying COVID-19 Decedents from Cause of Death Fields**

The death certificates that we rely on include text fields for primary cause of death, other conditions in the causal chain, and other significant conditions. We conduct text analysis of these fields to determine which deaths are likely to be caused by COVID-19. Table App-1 compares the COVID-19 counts we determine using text analysis to those from the ICD-10 codes included in death records, which are generated by personnel at the National Center for Health Statistics (NCHS) based on the text fields. Using ICD-10 codes produces many false negatives (deaths that we code as caused by COVID-19, but do not have COVID-19 coded as the primary cause of death, using ICD-10 code U07.1) and a smaller but still meaningful number of false positives (deaths that we code as not caused by COVID-19, but having U07.1 listed as the primary cause of death in the ICD-10 codes).

For Milwaukee County, our text-based measure counts 1,911 COVID-19 deaths during our sample period, whereas ICD-10 codes from NCHS identify only 1,214 COVID-19 deaths. For Wisconsin as a whole, the NCHS undercount is smaller; we count 12,595 COVID-19 deaths versus 11,512 using NCHS coding.

Most of the false negatives and false positives produced by the ICD-10 codes are not close cases. The NCHS coding is simply wrong, based on the text fields. Most of the false negatives involve COVID-19 deaths which were coded as B99 (other and unspecified infectious diseases). This coding error is largely limited to Milwaukee County; use of B99 in the rest of Wisconsin is rare. We speculate that the coder at NCHS responsible for Milwaukee death certificates did not realize there was a specific code for COVID-19 death. The false positives (deaths recorded with U07.1 as primary cause of death, that we coded as not due to COVID-19) typically lacked meaningful support from the text fields for COVID-19 as a cause of death. Reliance on the text fields reduces classification error, in which COVID deaths are misclassified as non-COVID or vice-versa, and thus improves the accuracy with which the CEMP measure controls for population health, as a predictor of COVID-19 mortality risk that is independent of vaccination status. We provide evidence on whether our approach undercounts COVID-19 deaths in Figures App-2 and App-3. Figure App-2, Panel A reports monthly natural non-COVID-19 and all natural deaths for Wisconsin for 2017 – June 2022. For the pandemic period, we also show predicted natural non-COVID deaths, based on linear extrapolation from 2017-2019 to the same calendar month during the pandemic period. Natural deaths (including COVID-19 deaths) show COVID-related peaks in late 2020 and late-2021-early 2022. Natural non-COVID-19 deaths do not have substantial corresponding spikes (beyond normal seasonal effects), which would be expected if COVID deaths were undercounted. Predicted non-COVID natural deaths (dashed line) are close to measured deaths, sometimes higher or lower, including during the peak COVID mortality periods. but with no obvious pattern.

Panel B is similar but limited to Milwaukee County for 2020-June, 2022. It shows predicted and actual natural non-COVID deaths, plus a 95% confidence interval (CI) around predicted deaths. Actual deaths are generally within the CI for predicted deaths, occasionally above or below,but with no consistent pattern.

Figure App-3 is similar to Figure App-2, Panel A, but provides an out-of-sample analysis for Indiana, for which we have similar mortality data. These figures provide evidence that our text-based coding of COVID-19 deaths does a good job of capturing actual COVID-19 deaths, and thus provides a reasonably reliable CEMP numerator and denominator.

The detailed coding we used to identify deaths as due to or probably due to COVID-19 is available from the authors on request. In brief, we counted as COVID-19 deaths those for which:

(i) COVID-19 or variants (such as SARS-2, SARS-COV-2, coronavirus) was listed as the principal cause of death; or

(ii) COVID-19 or variants were listed in the causal chain and the principal cause of death was likely to be caused by COVID-19 (for example, pneumonia, respiratory failure, hypoxia); or

(iii) COVID-19 or variants were listed in the causal chain and the principal cause of death was a known potential outcome of COVID-19 infection (for example, heart attack, stroke, sepsis) or involved underlying disease that was plausibly exacerbated by COVID-19 infection (for example, heart failure, COPD, dementia); or

(iv) COVID-19 was included in the "other significant conditions" field, but not in the causal chain fields, and (a) the principal cause of death was likely to be caused by COVID (group (ii) above); and (b) there was no entry in the causal chain fields indicating another likely cause of death.

We view reliability as high for categories (i) and (ii), and moderate for categories (iii) and (iv). The false positives (COVID-19 as principal cause of death based on ICD-10 codes) involve deaths for which the text fields do not fit in any of these four categories.

**Virus Variants and Periods of Dominance**

Accounting for the two-week lag we impose between dose administration and assumed effectiveness, the Alpha strain was dominant in the U.S. starting the week of March 14, 2021; the Delta variant was dominant beginning the week of July 4, 2021, and the Omicron variant has been dominant starting the week of Jan. 2, 2022.[2] These dominance periods correspond reasonably closely to the calendar quarters we use in text.

**Measuring Population by Month and Number of Vaccine Doses**

We construct a synthetic Milwaukee population by age-group and month as follows. We begin with American Community Survey (ACS) population estimates by age for 2020, which we assume apply to January 2020. The available age ranges in ACS are: 0-4; 5-9; 10-14; 15-17; 18-19; 20; 21; 22-24; 25-29; 30-34; 35-39; 40-44; 45-49; 50-54; 55-59; 60-61; 62-64; 65-66; 67-69; 70-74; 75-79; 80-84; and 85+. ACS provides only a single count for persons age 85+. Within each ACS age group we use the annual survival probabilities from NCHS by age and gender for 2018 (most recent year with available data) to divide the ACS population for that age group (in

---

[2] Source: https://www.gisaid.org/; see also https://covid.cdc.gov/covid-data-tracker/?utm_source=STAT+Newsletters&utm_campaign=059492f101-MR_COPY_01&utm_medium=email&utm_term=0_8cab1d7961-059492f101-153972538#variant-proportions

this example, ages 75-79) into an estimated number of persons age 75, 76, 77, 78, and 79. For the age 85+ group, we assume a maximum age of 100. Within each year of age, we assume birth month is random, so that approximately 1/12 of the population will be born in January, approximately 1/12 in February, and so on. We then roll forward a month at a time during the sample period. Thus, going from January to February 2020, everyone gets one month older and some (those assigned a January birth month) will be one year older. This gives us a starting synthetic population (before allowing for death) by year-month of age, for each calendar month.[3]

We next adjust this initial synthetic population for deaths. We ignore in- and out-migration, for which we lack data. From our mortality data, we have age at death in years; we assign month of birth randomly and compute age at death in months. In each calendar month we subtract the decedents from the synthetic population, to obtain a synthetic alive population by year-month of age for each calendar month. In a small number of instances, for very advanced ages, this process results in small negative population counts in (year-month of age)*calendar month cells; we convert negative values to zero.

### Time Periods and Booster Rollout

Boosters were first authorized by the Centers for Disease Control and Prevention (CDC) on September 24, 2021, initially for ages 60+ and healthcare workers and others in high -risk occupations. The CDC authorized boosters for all adults on November 19, 2021, but recommended them only for ages 50+.[4] Finally, on November 29, 2021, the CDC recommended boosters for all adults.[5] The FDA and CDC authorized second boosters for use by persons aged 50+ on March 29, 2022. These agencies authorized bivalent boosters (including an Omicron-specific component for all ages on Sept. 1, 2022**.**

### Details on Younger, Fully Vaccinated COVID-19 Decedents

Through June 30, 2022, only one two-dose vaccinated person died of COVID-19 in the age range from 18-49: An individual with sickle cell anemia, hemochromatosis, and pulmonary hypertension; vaccinated with Pfizer in April and May 2021 eight months after the second dose, during the Omicron period. For ages 50-59, there were 8 deaths through June 30, 2022, of two-dose vaccinees (4 Pfizer and 4 Moderna). A majority had major comorbidities, but several did not.

### Vaccination Counts and Rates

We obtained data on vaccinated individuals in Milwaukee County, including date of each vaccine dose, and vaccine type for each dose from the Wisconsin Immunization Registry. Table App-2 provides summary data on vaccinated adults in Milwaukee, how many doses they received, and which vaccine they received.

In Figure App-4, we report overall vaccination rates for Milwaukee County adults over Panel A, provides information on "full vaccination" rates (two mRNA doses or one J&J dose) by

---

3 We do not increase years of age to greater than 100 during this process.

4 CDC Press Release (Nov. 19, 2021); Mandavilli (2021).

5 CDC Press Release (Nov. 29, 2021).

age range over time. Vaccine uptake was faster and more complete among those aged 60+ and highest for ages 60-79, consistent with national trends. Panel B provides information on receipt of a third dose, generally a booster dose following two-dose mRNA vaccination. Three-dose percentages (conditional on receiving two doses) rise with age, but are similar for ages 60-79 and 80+.

**Confidence Intervals for Text Table 2**

Table App-3 provides 95% confidence intervals (CIs) for the relative mortality risk (RMR) values reported in text Table 2.

**Additional Covariates for Multivariable Logit Model**

In the multivariable logistic analysis reported in text, we used limited covariates to preserve degrees of freedom: age, $age^2$, gender, and (days since last vaccine dose - 30 days). In Table App-4, we repeat this analysis with the following additional covariates: race/ethnicity (White vs. minority; education (high school or less vs. some college or more), and zip-SES. Results are similar to the model reported in the text.

The covariates for education and zip-SES, which are known from other work to predict COVID-19 mortality, have little effect on CEMP-based estimates of RMR. This is consistent with the CEMP denominator already capturing must of the effect of these covariates on mortality.

**Defining the Immune-Compromised**

In the results in text, we exclude persons known to be immune-compromised, defined as: (i) decedents whose death records indicate a solid organ transplant; (ii) decedents whose death records state that they were immune-compromised, had rheumatic disease, or Crohn's disease; and (iii) persons who received a third vaccine dose on or before September 24, 2021, the first date when a third dose was authorized for the non-immune-compromised. The standard CDC recommendation for full vaccination included a third "primary" dose; for these persons, the first booster dose would be a fourth dose. The booster studies by Arbel et. al (2021) and Bar-On et. al (2021) also exclude early third-dose recipients.

We report results without excluding the immune-compromised in Table App-5. As expected, RMR values versus the unvaccinated are generally somewhat higher, but results for the Pfizer/Moderna ratio are similar to those reported in the text.

Conversely, our approach to excluding the immune-compromised may undercount them, and thus overstate RMR. Therefore, in Table App-6, as a further robustness check, we also exclude all decedents whose death certificates refer to cancer, many of whom will be immune-compromised to varying degrees due to cancer treatment. RMR values versus the unvaccinated and Pfizer/Moderna ratio estimates are similar to those reported in the text.

**Lag Period Between Vaccine Administration and Effectiveness Against Death**

It is common, based on evidence on immune response to vaccination, to allow for a lag between when a vaccine dose is administered and when it is treated as effective. Most studies use 7-14 day periods. We use a 30-day lag to both allow the vaccine to reach full effect, and capture

typical lags from infection to death. An extreme example of failure to allow for lagged effects is Andrews et. al (2022), discussed in text above, which reported 1.3% RMR for a Pfizer booster against death, but measured death over a very limited period of 14-34 days after booster receipt.[6] Thus, this study counted as COVID decedents only persons infected at least 14 days after booster, who died within 34 days after booster. This would be expected to produce severe downward bias in estimating RMR.

**Results by Gender**

In Table App-7, we present results, similar to text Table 2, separately for men and women. Results by gender are generally similar to the both-gender results in the text, for both RMRs and the Pfizer/Moderna ratio. Two-dose RMR versus the unvaccinated for Pfizer is (20%, 41%, 77%) across the three sample periods for men, versus (32%, 46%, 37%) for women; note the outlier result for men in the Omicron period, which is present for both ages 18-59 and ages 60+. For Moderna, two-dose RMR versus the unvaccinated is (20%, 14%, 28%) for men versus (12%, 22%, 16%) for women. Thus, the outlier result for men in the Omicron period is limited to Pfizer vaccinees. The male-vs-female difference in RMRs warrant further study as additional data for the Omicron period becomes available.

Three-dose RMR versus the unvaccinated for Pfizer vaccinees for the two available periods is (0%, 13%) for men versus (12%, 13%) for women. The Omicron period differences in male-vs-female RMRs is thus present only for two-dose vaccinees.

**Results for Whites versus Other Racial/Ethnic Groups**

In Table App-8, we present results, similar to text Table 2, separately for non-Hispanic Whites, and minorities (all other persons). Sample size is too small to permit further decomposition of the results by minority group. Although the small number of COVID-19 deaths among two- and three-dose recipients lead to noisy estimates, there is no evidence of differences between Whites and other racial/ethnic groups in RMRs versus he unvaccinated or in or the Pfizer/Moderna ratio.

**Non-Covid Natural Mortality in 2019 as Predictor of COVID-19 Mortality in Pre-Vaccine Period**

Figure App-5shows the correlation in Milwaukee County between natural mortality in April-December 2019 (pre-COVID period) and COVID-19 mortality in April-December 2020 (COVID period, but pre-vaccine), for population groups defined by age (groups are 18-39, 40-49, 50-59, 60-69, 70-79, 80-89, and 90+, gender, race/ethnicity, and zip-SES. The Pearson correlation coefficient is 0.94, consistent with non-COVID natural mortality rates strongly predicting COVID mortality rates for unvaccinated persons.

Further validation comes from the multivariate regression analysis in text Table 3 and in Table App-4, in which RMR estimates within groups defined solely by age are similar to multivariate estimates that adjust for other factors associated with COVID-19 mortality. This suggests that the CEMP measure already controls well for population health.

---

[6] Andrews et. al (2022).

### Evidence on Selection Effects from Non-Covid-NMRs

Selection effects can arise from several sources. First, the vaccinated can be in better health than the unvaccinated (Atanasov et. al, 2022; Cohn et. al, 2022). Second, among the vaccinated, there will be timing differences in when vaccination occurred, some deriving from the rollout of vaccines in early 2021, with healthcare workers, the elderly, and those with major comorbidities generally eligible earlier than other persons. Within any eligible group, the more health conscious, and thus healthier, might get vaccinated earlier. Third, the Pfizer and Moderna vaccines became available at different times, which Pfizer generally easier to find earlier in 2021. These differences in vaccination timing will interact with waning VE over time since vaccination, and with different speeds of waning, where prior studies provide evidence that Pfizer wanes more rapidly than Moderna (see the review by Black and Thaw, 2022

Table App-9 provides data on non-COVID-19 natural mortality rates (Non-Covid-NMRs) by time period, age group, and vaccination status. This table provides evidence that selection effects are important. Vaccinees were healthier than the unvaccinated at all ages, shown in the rows for Non-Covid-NMRs versus the unvaccinated. In addition, Pfizer vaccinees were healthier than Moderna vaccinees. In unreported results, we find that Pfizer vaccinees were vaccinated somewhat earlier than Moderna vaccinees, which may proxy for greater health consciousness, leading to becoming vaccinated as soon as vaccines were available.

### Additional Evidence on Selection Effects: CEMP vs. Covid-PFR as Outcome

One way to assess the importance of using CEMP to control for selection effects is to rerun our analysis using the same age groups and time periods, but using as the outcome Covid-PFR instead of CEMP. This outcome measure does not control for selection effects in who gets vaccinated. Table App-10 provides results for RMRs and the Pfizer/Moderna ratio using the Covid-PFR as the outcome. These RMRs are more comparable to other studies, many of which do not effectively control for selection effects. As expected, given the selection effects shown in Table App-10, RMRs for the vaccinated vs, the unvaccinated are substantially lower than those reported in the text, and closer to those reported in other studies. For Pfizer vaccinees age 60+, two-dose RMR versus the unvaccinated is (9%, 19%, 31%) across the three sample periods using Covid-PFR as the outcome, versus (35%, 44%, 56%) in text Table 2 (with CEMP as the outcome); and three-dose RMR versus the unvaccinated for the two available time periods is (5.3%, 2.4%) using Covid-PFR as the outcome versus (6.3%, 13.1%) in text Table 2.

The Pfizer/Moderna ratio is also much lower with Covid-PFR as the outcome, and not statistically different from 100%. Across the three time periods, this ratio is (108%, 129%, 148%) with Covid-PFR as the outcome, versus (229%, 240%, 254%) with CEMP as the outcome.

### Reporting Accuracy for COVID-19 Deaths and Excess Non-COVID-19 Natural Mortality

To the extent that our counts of COVID-19 deaths, although higher than NCHS counts, are still undercounts, this should show up in measures of excess non-COVID natural deaths, defined as non-COVID-19 natural deaths, minus predicted levels based on extrapolation of mortality trends from the pre-pandemic period. Undercounting of COVID-19 deaths would lead to excess deaths generally being positive during the pandemic periods, and spiking during the periods when measured COVID-19 mortality spikes. In text Figure 2, we reported data for Wisconsin, and found

no evidence of excess non-COVID natural deaths. In Figure App-3, we show a similar figure for Indiana, a nearby state where we also have access to detailed mortality records

In this figure, there is no evidence for a substantial number of excess non-COVID-19 natural deaths. The predicted line is above the actual line roughly as often as below it. Moreover, there is no tendency for the actual-minus-predicted gap to be large during period of high COVID-19 mortality.

Another possibility, on which Figure App-3 also provide evidence, is that prior COVID-19 infection will lead to higher future deaths from natural causes, not directly linkable to the prior infection. Higher post-infection mortality would predict positive actual-minus-predicted non-COVID-19 natural mortality generally, not limited to periods of high COVID-19 mortality. There is evidence from other research of excess cardiovascular risk for a limited period of time following infection (Rezel-Potts et. al, 2022). However, the extent of excess deaths is not known. Any excess deaths for our sample are too small to be apparent from Figure App-2.

### Additional References for Online Appendix (not cited in the text)

Griffin, Jennifer B, Meredith Haddix, Phoebe Danza, Rebecca Fisher, Tae Hee Koo, Elizabeth Traub, Prabhu Gounder, Claire Jarashow, and Sharon Balter (2021), SARS-CoV-2 Infections and Hospitalizations Among Persons Aged ≥16 Years, by Vaccination Status — Los Angeles County, California, May 1–July 25, 2021, *Morbidity and Mortality Weekly Report* 70(34), 1170-1176.

Mandavilli, Apoorva (2021), C.D.C. Endorses Covid Vaccine Booster Shots for All Adults, *New York Times* (Nov. 19).

Rezel-Potts, Emma, Abdel Douiri, Xiaohui Sun, Phillip J. Chowienczyk, Ajay M. Shah, and Martin C. Gulliford (2022), Cardiometabolic outcomes up to 12 months after COVID-19 infection. A matched cohort study in the UK, *PLoS*, https://doi.org/10.1371/journal.pmed.1004052.

## Table S1. Comparison of COVID-19 deaths per text analysis, to ICD-10 codes

Table shows counts, for April 1, 2020, through June 30, 2022, 2022, of COVID-19 deaths determined from our text analysis to COVID-19 deaths, determined using ICD-10 codes (the code for COVID-19 cause of death is U07.1). ICD-10 codes are generated by NCHS, based on the text fields in the death certificates. Deaths from natural causes are all deaths except those due to accident, homicide, or suicide. **Panel A.** Milwaukee County. **Panel B.** Wisconsin (statewide**.**

**Panel A. Milwaukee County**

| **Milwaukee County** | **COVID-19 by Text Analysis** | **non-COVID-19 by Text Analysis** | **Total** |
|---|---|---|---|
| COVID-19 by ICD-10 Code | 1,351 | 18 | **1,369** |
| non-COVID-19 by ICD-10 Code | 583 | 19,831 | **20,414** |
| Missing ICD-10 Code | 0 | 46 | **46** |
| **Total** | **1,934** | **19,895** | **21,829** |
| From Natural Causes | | | 18,690 |

**Panel B. Wisconsin (statewide)**

| **Wisconsin (statewide)** | **COVID-19 by Text Analysis** | **non-COVID-19 by Text Analysis** | **Total** |
|---|---|---|---|
| COVID-19 by ICD-10 Code | 11,303 | 209 | **11,512** |
| non-COVID-19 by ICD-10 Code | 1,292 | 122,494 | **123,786** |
| Missing ICD-10 Code | 0 | 188 | **188** |
| **Total** | **12,595** | **122,891** | **135,486** |
| From Natural Causes | | | 122,870 |

## Table S2. Summary Statistics on Vaccination Status and Vaccine Type

Table provides summary information on vaccine doses for adults vaccinated in Milwaukee County, through June 30 31, 2022. Vaccine type for fully vaccinated persons is based on first two doses. Includes immune-compromised persons. Number of never vaccinated inferred from the population total and population of vaccinated. Numbers exclude immune-compromised persons. Natural deaths are over April 1, 2021, through June 30, 2022 (shorter period than in Table App-1).

| | | **Population** | | **Natural Deaths** | |
|---|---|---|---|---|---|
| Number of Doses | Vaccine Type | **Number** | **%** | **Number** | **%** |
| Exactly 1 | Moderna | 11,264 | 1.6% | 278 | 2.9% |
| | Pfizer | 21,436 | 3.0% | 312 | 3.3% |
| | J&J | 19,000 | 2.6% | 271 | 2.9% |
| Exactly 2 | Moderna Only | 64,186 | 8.9% | 1,876 | 19.7% |
| | Pfizer Only | 124,879 | 17.3% | 1,358 | 14.3% |
| | J&J Only | 2,189 | 0.3% | 9 | 0.1% |
| | Mixed mRNA | 1,018 | 0.1% | 28 | 0.3% |
| | Mixed J&J and mRNA | 12,890 | 1.8% | 45 | 0.5% |
| 3 or more | Moderna Only | 95,946 | 13.3% | 772 | 8.1% |
| | Pfizer Only | 157,077 | 21.8% | 601 | 6.3% |
| | Mixed mRNA | 26,002 | 3.6% | 120 | 1.3% |
| | Mixed J&J and mRNA | 422 | 0.1% | 1 | 0.0% |
| **Vaccinated and not immunocompromised** | | 536,309 | 74.3% | 5,671 | 59.7% |
| **Received 2+ Pfizer or 2+ Moderna only** | | 442,008 | 61.3% | 4,607 | 48.5% |
| **Received 3+ Pfizer or 3+ Moderna only** | | 253,023 | 35.1% | 1,373 | 14.4% |
| **Immunocompromised** | | 5,843 | 0.8% | 289 | 3.0% |
| **Never vaccinated** | | 179,366 | 24.9% | 3,545 | 37.3% |
| **Total for Milwaukee Adult Population (age 18+)** | | 721,518 | | 9,505 | |

## Table S3. Confidence Intervals for Pfizer/Moderna Ratio in Text Table 2

Table reports 95% confidence intervals (CIs) and p-values for the Pfizer/Moderna Ratio estimates reported for ages 18-59 and 60+ in text Table 2. The estimates are from logistic regressions (one for each time period and vaccination group – 2 doses or 3 doses), with an outcome variable equal to 1 for decedents who died of COVID-19 and 0 for decedents who died of other natural causes. The regressions use as a predictor an indicator equal to 1 for Pfizer and 0 for Moderna. The table also reports point estimates and CIs for two-dose vaccinees for the full sample period (April 2021 – June 2022), and for booster recipients for the full booster-available period (October 2021 – June 2022) for two-dose vaccinees Boldface indicates significant differences at p = .05 or better.

| | | **Two Doses** | | | **Three Doses** | | |
|---|---|---|---|---|---|---|---|
| | | **Pfizer/Moderna Ratio** | | | **Pfizer/Moderna Ratio** | | |
| Age Group | Time Period | Estimate | P-value | 95% CI | Estimate | P-value | 95% CI |
| 18-59 | Apr-Sep 2021 | No Moderna Deaths | | | Boosters not available | | |
| | Oct-Dec 2021 | 52.17% | 0.649 | [3.2%, 858.8%] | No Moderna or Pfizer Deaths | | |
| | Jan-Jun 2022 | 88.26% | 0.874 | [18.9%, 412.4%] | No Moderna or Pfizer Deaths | | |
| | Apr 2021-Jun 2022 | 96.04% | 0.951 | [26.8%, 344.4%] | No Moderna or Pfizer Deaths | | |
| 60+ | Apr-Sep 2021 | **229.12%** | 0.032 | [107.5%, 488.4%] | Boosters not available | | |
| | Oct-Dec 2021 | **240.29%** | 0.001 | [144.9%, 398.5%] | 47.22% | 0.545 | [4.2%, 536.8%] |
| | Jan-Jun 2022 | **254.35%** | 0.002 | [140.9%, 459.0%] | 149.73% | 0.319 | [67.7%, 331.0%] |
| | Apr 2021-Jun 2022 | **248.08%** | 0.000 | [176.7%, 348.3%] | | not relevant | |
| | Oct 2021-Jun 2022 | | Not relevant | | 133.88% | 0.445 | [63.3%, 283.2%] |

## Table S4. Multivariate Logit Model with Additional Covariates

Sample is same as text Table 3. Table presents odds ratios are for Pfizer vaccinee mortality relative to Moderna vaccinees (P/M ratio), from logit model of Prob(Covid-19 Death) = f(received Pfizer (Moderna is baseline), with controls for age, $age^2$, gender, and (days since last vaccine dose - 30 days) and the following additional covariates, not included in text Table 3: : race/ethnicity (White vs. non-White); education (high school or less vs. some college or more), and zip-SES. Statistically significantly greater than 100% odds ratios (at 5% level or better) in **boldface**. For two doses, ages 18-59, Oct-Dec 2021, there are two few deaths to run the regression with additional covariates, beyond those in text Table 3.

| | | Two doses | | | Three doses | | |
|---|---|---|---|---|---|---|---|
| Sample | Period | P/M ratio | P-Value | 95 CI | P/M ratio | P-Value | 95 CI |
| 18-59 | Apr-Sep 2021 | No deaths | | NA | No booster | | |
| | Oct-Dec 2021 | Insufficient observations | | NA | No deaths | NA | NA |
| | Jan-Jun 2022 | 84.1% | 0.834 | [16.7%, 424.4%] | No deaths | NA | NA |
| | Jan 2021-June 2022 | 106.5% | 0.927 | [27.9%, 405.7%] | No deaths | NA | NA |
| 60+ | Apr-Sep 2021 | **266.1%** | **0.017** | **[119.4%, 593.0%]** | No booster | | |
| | Oct-Dec 2021 | **250.4%** | **0.001** | **[144.2%, 435.1%]** | 16.8% | 0.420 | [0.2%, 1283.4%] |
| | Jan-Jun 2022 | **226.2%** | **0.011** | **[121.0%, 422.8%]** | 147.1% | 0.375 | [62.7%, 345.1%] |
| | Jan 2021-June 2022 | **250.4%** | **0.000** | **[175.9%, 356.4%]** | 130.5% | 0.513 | [58.8%, 289.8%] |

## Table S5. RMRs and Pfizer/Moderna Ratio by Age Group and Time Period, Including Immune-Compromised

Table is similar to text Table 2, but sample includes immune-compromised persons.

| | | Apr-Sep 2021 | | | Oct-Dec 2021 | | | | | Jan-Jun 2022 | | | | |
|---|---|---|---|---|---|---|---|---|---|---|---|---|---|---|
| Age Bracket | Death | Unvax | M2 | P2 | Unvax | M2 | P2 | M3 | P3 | Unvax | M2 | P2 | M3 | P3 |
| 18-39 | Covid Deaths | 9 | 0 | 0 | 18 | 0 | 0 | 0 | 0 | 4 | 0 | 1 | 0 | 0 |
| | Non-Covid Natural Deaths | 64 | 5 | 5 | 26 | 5 | 6 | 0 | 1 | 32 | 5 | 13 | 2 | 2 |
| | CEMP | 14.1% | 0.0% | 0.0% | 69.2% | 0.0% | 0.0% | NA | 0.0% | 12.5% | 0.0% | 7.7% | 0.0% | 0.0% |
| | RMR to unvax | | 0.0% | 0.0% | | 0.0% | 0.0% | NA | 0.0% | | 0.0% | 61.5% | 0.0% | 0.0% |
| | Pfizer RMR to Moderna | | | NA | | | NA | | NA | | | NA | | NA |
| 40-59 | Covid Deaths | 40 | 1 | 1 | 58 | 1 | 2 | 0 | 0 | 23 | 5 | 3 | 1 | 1 |
| | Non-Covid Natural Deaths | 263 | 29 | 54 | 103 | 31 | 63 | 2 | 6 | 180 | 43 | 59 | 38 | 45 |
| | CEMP | 15.2% | 3.4% | 1.9% | 56.3% | 3.2% | 3.2% | 0.0% | 0.0% | 12.8% | 11.6% | 5.1% | 2.6% | 2.2% |
| | RMR to unvax | | 22.7% | 12.2% | | 5.7% | 5.6% | 0.0% | 0.0% | | 91.0% | 39.8% | 20.6% | 17.4% |
| | Pfizer RMR to Moderna | | | 53.7% | | | 98.4% | | NA | | | 43.7% | | 84.4% |
| 60-79 | Covid Deaths | 76 | 5 | 12 | 97 | 14 | 21 | 4 | 1 | 92 | 9 | 20 | 5 | 12 |
| | Non-Covid Natural Deaths | 675 | 300 | 277 | 270 | 203 | 180 | 21 | 43 | 418 | 221 | 189 | 258 | 286 |
| | CEMP | 11.3% | 1.7% | 4.3% | 35.9% | 6.9% | 11.7% | 19.0% | 2.3% | 22.0% | 4.1% | 10.6% | 1.9% | 4.2% |
| | RMR to unvax | | 14.8% | 38.5% | | 19.2% | 32.5% | 53.0% | 6.5% | | 18.5% | 48.1% | 8.8% | 19.1% |
| | Pfizer RMR to Moderna | | | 259.9% | | | 169.2% | | 12.2% | | | 259.8% | | 216.5% |
| 80+ | Covid Deaths | 33 | 9 | 7 | 50 | 15 | 23 | 1 | 2 | 57 | 11 | 16 | 9 | 7 |
| | Non-Covid Natural Deaths | 466 | 539 | 216 | 227 | 322 | 130 | 41 | 29 | 309 | 190 | 118 | 453 | 276 |
| | CEMP | 7.1% | 1.7% | 3.2% | 22.0% | 4.7% | 17.7% | 2.4% | 6.9% | 18.4% | 5.8% | 13.6% | 2.0% | 2.5% |
| | RMR to unvax | | 23.6% | 45.8% | | 21.1% | 80.3% | 11.1% | 31.3% | | 31.4% | 73.5% | 10.8% | 13.7% |
| | Pfizer RMR to Moderna | | | 194.1% | | | 379.8% | | 282.8% | | | 234.2% | | 127.7% |
| 18-59 | Covid Deaths | 49 | 1 | 1 | 76 | 1 | 2 | 0 | 0 | 27 | 5 | 4 | 1 | 1 |
| | Non-Covid Natural Deaths | 327 | 34 | 59 | 129 | 36 | 69 | 2 | 7 | 212 | 48 | 72 | 40 | 47 |
| | CEMP | 15.0% | 2.9% | 1.7% | 58.9% | 2.8% | 2.9% | 0.0% | 0.0% | 12.7% | 10.4% | 5.6% | 2.5% | 2.1% |
| | RMR to unvax | | 19.6% | 11.3% | | 4.7% | 4.9% | 0.0% | 0.0% | | 81.8% | 43.6% | 19.6% | 16.7% |
| | Pfizer RMR to Moderna | | | 57.6% | | | 104.3% | | NA | | | 53.3% | | 85.1% |
| 60+ | Covid Deaths | 109 | 14 | 19 | 147 | 29 | 44 | 5 | 3 | 149 | 20 | 36 | 14 | 19 |
| | Non-Covid Natural Deaths | 1,141 | 839 | 493 | 497 | 525 | 310 | 62 | 72 | 727 | 411 | 307 | 711 | 562 |
| | CEMP | 9.6% | 1.7% | 3.9% | 29.6% | 5.5% | 14.2% | 8.1% | 4.2% | 20.5% | 4.9% | 11.7% | 2.0% | 3.4% |
| | RMR to unvax | | 17.5% | 40.3% | | 18.7% | 48.0% | 27.3% | 14.1% | | 23.7% | 57.2% | 9.6% | 16.5% |
| | Pfizer RMR to Moderna | | | 231.0% | | | 257.0% | | 51.7% | | | 241.0% | | 171.7% |

## Table S6. RMRs and Pfizer/Moderna Ratio by Age Group and Time Period

Table is similar to text Table 2, but sample excludes both known immune-compromised persons and persons with cancer indicated in death certificates, many of whom will be immune-compromised.

| | | Apr-Sep 2021 | | | Oct-Dec 2021 | | | | | Jan-Jun 2022 | | | | |
|---|---|---|---|---|---|---|---|---|---|---|---|---|---|---|
| Age Bracket | Death | Unvax | M2 | P2 | Unvax | M2 | P2 | M3 | P3 | Unvax | M2 | P2 | M3 | P3 |
| 18-39 | Covid Deaths | 9 | 0 | 0 | 16 | 0 | 0 | 0 | 0 | 4 | 0 | 1 | 0 | 0 |
| | Non-Covid Natural Deaths | 56 | 5 | 3 | 21 | 2 | 6 | 0 | 0 | 28 | 4 | 9 | 1 | 2 |
| | CEMP | 16.1% | 0.0% | 0.0% | 76.2% | 0.0% | 0.0% | NA | NA | 14.3% | 0.0% | 11.1% | 0.0% | 0.0% |
| | RMR to unvax | | 0.0% | 0.0% | | 0.0% | 0.0% | NA | NA | | 0.0% | 77.8% | 0.0% | 0.0% |
| | Pfizer RMR to Moderna | | | NA | | | NA | | NA | | | NA | | NA |
| 40-59 | Covid Deaths | 35 | 0 | 1 | 57 | 0 | 1 | 0 | 0 | 23 | 3 | 3 | 0 | 0 |
| | Non-Covid Natural Deaths | 187 | 25 | 42 | 80 | 24 | 48 | 0 | 2 | 133 | 35 | 39 | 27 | 31 |
| | CEMP | 18.7% | 0.0% | 2.4% | 71.3% | 0.0% | 2.1% | NA | 0.0% | 17.3% | 8.6% | 7.7% | 0.0% | 0.0% |
| | RMR to unvax | | 0.0% | 12.7% | | 0.0% | 2.9% | NA | 0.0% | | 49.6% | 44.5% | 0.0% | 0.0% |
| | Pfizer RMR to Moderna | | | NA | | | NA | | NA | | | 89.7% | | NA |
| 60-79 | Covid Deaths | 69 | 4 | 7 | 84 | 11 | 16 | 1 | 0 | 82 | 6 | 13 | 3 | 5 |
| | Non-Covid Natural Deaths | 457 | 214 | 175 | 191 | 157 | 121 | 11 | 20 | 298 | 164 | 128 | 172 | 165 |
| | CEMP | 15.1% | 1.9% | 4.0% | 44.0% | 7.0% | 13.2% | 9.1% | 0.0% | 27.5% | 3.7% | 10.2% | 1.7% | 3.0% |
| | RMR to unvax | | 12.4% | 26.5% | | 15.9% | 30.1% | 20.7% | 0.0% | | 13.3% | 36.9% | 6.3% | 11.0% |
| | Pfizer RMR to Moderna | | | 214.0% | | | 188.7% | | 0.0% | | | 277.6% | | 173.7% |
| 80+ | Covid Deaths | 29 | 8 | 6 | 42 | 15 | 18 | 1 | 0 | 52 | 10 | 13 | 8 | 6 |
| | Non-Covid Natural Deaths | 369 | 454 | 160 | 198 | 276 | 91 | 33 | 17 | 260 | 169 | 101 | 398 | 201 |
| | CEMP | 7.9% | 1.8% | 3.8% | 21.2% | 5.4% | 19.8% | 3.0% | 0.0% | 20.0% | 5.9% | 12.9% | 2.0% | 3.0% |
| | RMR to unvax | | 22.4% | 47.7% | | 25.6% | 93.2% | 14.3% | 0.0% | | 29.6% | 64.4% | 10.1% | 14.9% |
| | Pfizer RMR to Moderna | | | 212.8% | | | 364.0% | | 0.0% | | | 217.5% | | 148.5% |
| 18-59 | Covid Deaths | 44 | 0 | 1 | 73 | 0 | 1 | 0 | 0 | 27 | 3 | 4 | 0 | 0 |
| | Non-Covid Natural Deaths | 243 | 30 | 45 | 101 | 26 | 54 | 0 | 2 | 161 | 39 | 48 | 28 | 33 |
| | CEMP | 18.1% | 0.0% | 2.2% | 72.3% | 0.0% | 1.9% | NA | 0.0% | 16.8% | 7.7% | 8.3% | 0.0% | 0.0% |
| | RMR to unvax | | 0.0% | 12.3% | | 0.0% | 2.6% | NA | 0.0% | | 45.9% | 49.7% | 0.0% | 0.0% |
| | Pfizer RMR to Moderna | | | NA | | | NA | | NA | | | 108.3% | | NA |
| 60+ | Covid Deaths | 98 | 12 | 13 | 126 | 26 | 34 | 2 | 0 | 134 | 16 | 26 | 11 | 11 |
| | Non-Covid Natural Deaths | 826 | 668 | 335 | 389 | 433 | 212 | 44 | 37 | 558 | 333 | 229 | 570 | 366 |
| | CEMP | 11.9% | 1.8% | 3.9% | 32.4% | 6.0% | 16.0% | 4.5% | 0.0% | 24.0% | 4.8% | 11.4% | 1.9% | 3.0% |
| | RMR to unvax | | 15.1% | 32.7% | | 18.5% | 49.5% | 14.0% | 0.0% | | 20.0% | 47.3% | 8.0% | 12.5% |
| | Pfizer RMR to Moderna | | | 216.0% | | | 267.1% | | 0.0% | | | 236.3% | | 155.7% |

## Table S7. RMRs and Pfizer/Moderna Ratio by Gender

Table and sample are same as text Table 2, but we show results separately for men (**Panel A**) and women (**Panel B**)

**Panel A. Male**

| | | Apr-Sep 2021 | | | Oct-Dec 2021 | | | | | Jan-Jun 2022 | | | | |
|---|---|---|---|---|---|---|---|---|---|---|---|---|---|---|
| Age Bracket | Death | Unvax | M2 | P2 | Unvax | M2 | P2 | M3 | P3 | Unvax | M2 | P2 | M3 | P3 |
| 18-39 | Covid Deaths | 6 | 0 | 0 | 8 | 0 | 0 | 0 | 0 | 2 | 0 | 0 | 0 | 0 |
| | Non-Covid Natural Deaths | 35 | 2 | 4 | 16 | 3 | 3 | 0 | 0 | 19 | 3 | 9 | 1 | 2 |
| | CEMP | 17.1% | 0.0% | 0.0% | 50.0% | 0.0% | 0.0% | NA | NA | 10.5% | 0.0% | 0.0% | 0.0% | 0.0% |
| | RMR to unvax | | 0.0% | 0.0% | | 0.0% | 0.0% | NA | NA | | 0.0% | 0.0% | 0.0% | 0.0% |
| | Pfizer RMR to Moderna | | | NA | | | NA | | NA | | | NA | | NA |
| 40-59 | Covid Deaths | 23 | 0 | 0 | 30 | 1 | 0 | 0 | 0 | 10 | 2 | 2 | 0 | 0 |
| | Non-Covid Natural Deaths | 147 | 15 | 26 | 55 | 15 | 35 | 0 | 1 | 122 | 24 | 29 | 18 | 29 |
| | CEMP | 15.6% | 0.0% | 0.0% | 54.5% | 6.7% | 0.0% | NA | 0.0% | 8.2% | 8.3% | 6.9% | 0.0% | 0.0% |
| | RMR to unvax | | 0.0% | 0.0% | | 12.2% | 0.0% | NA | 0.0% | | 101.7% | 84.1% | 0.0% | 0.0% |
| | Pfizer RMR to Moderna | | | NA | | | 0.0% | | NA | | | 82.8% | | NA |
| 60-79 | Covid Deaths | 35 | 3 | 3 | 44 | 5 | 10 | 0 | 0 | 45 | 4 | 13 | 1 | 2 |
| | Non-Covid Natural Deaths | 382 | 144 | 141 | 141 | 98 | 97 | 6 | 14 | 246 | 112 | 99 | 122 | 137 |
| | CEMP | 9.2% | 2.1% | 2.1% | 31.2% | 5.1% | 10.3% | 0.0% | 0.0% | 18.3% | 3.6% | 13.1% | 0.8% | 1.5% |
| | RMR to unvax | | 22.7% | 23.2% | | 16.3% | 33.0% | 0.0% | 0.0% | | 19.5% | 71.8% | 4.5% | 8.0% |
| | Pfizer RMR to Moderna | | | 102.1% | | | 202.1% | | NA | | | 367.7% | | 178.1% |
| 80+ | Covid Deaths | 17 | 3 | 5 | 32 | 5 | 12 | 1 | 0 | 32 | 7 | 9 | 5 | 5 |
| | Non-Covid Natural Deaths | 183 | 182 | 85 | 85 | 109 | 61 | 15 | 8 | 127 | 76 | 40 | 130 | 124 |
| | CEMP | 9.3% | 1.6% | 5.9% | 37.6% | 4.6% | 19.7% | 6.7% | 0.0% | 25.2% | 9.2% | 22.5% | 3.8% | 4.0% |
| | RMR to unvax | | 17.7% | 63.3% | | 12.2% | 52.3% | 17.7% | 0.0% | | 36.6% | 89.3% | 15.3% | 16.0% |
| | Pfizer RMR to Moderna | | | 356.9% | | | 428.9% | | 0.0% | | | 244.3% | | 104.8% |
| 18-59 | Covid Deaths | 29 | 0 | 0 | 38 | 1 | 0 | 0 | 0 | 12 | 2 | 2 | 0 | 0 |
| | Non-Covid Natural Deaths | 182 | 17 | 30 | 71 | 18 | 38 | 0 | 1 | 141 | 27 | 38 | 19 | 31 |
| | CEMP | 15.9% | 0.0% | 0.0% | 53.5% | 5.6% | 0.0% | NA | 0.0% | 8.5% | 7.4% | 5.3% | 0.0% | 0.0% |
| | RMR to unvax | | 0.0% | 0.0% | | 10.4% | 0.0% | NA | 0.0% | | 87.0% | 61.8% | 0.0% | 0.0% |
| | Pfizer RMR to Moderna | | | NA | | | 0.0% | | NA | | | 71.1% | | NA |
| 60+ | Covid Deaths | 52 | 6 | 8 | 76 | 10 | 22 | 1 | 0 | 77 | 11 | 22 | 6 | 7 |
| | Non-Covid Natural Deaths | 565 | 326 | 226 | 226 | 207 | 158 | 21 | 22 | 373 | 188 | 139 | 252 | 261 |
| | CEMP | 9.2% | 1.8% | 3.5% | 33.6% | 4.8% | 13.9% | 4.8% | 0.0% | 20.6% | 5.9% | 15.8% | 2.4% | 2.7% |
| | RMR to unvax | | 20.0% | 38.5% | | 14.4% | 41.4% | 14.2% | 0.0% | | 28.3% | 76.7% | 11.5% | 13.0% |
| | Pfizer RMR to Moderna | | | 192.3% | | | 288.2% | | 0.0% | | | 270.5% | | 112.6% |

**Panel B. Female**

| | | Apr-Sep 2021 | | | Oct-Dec 2021 | | | | | Jan-Jun 2022 | | | | |
|---|---|---|---|---|---|---|---|---|---|---|---|---|---|---|
| Age Bracket | Death | Unvax | M2 | P2 | Unvax | M2 | P2 | M3 | P3 | Unvax | M2 | P2 | M3 | P3 |
| 18-39 | Covid Deaths | 3 | 0 | 0 | 8 | 0 | 0 | 0 | 0 | 2 | 0 | 1 | 0 | 0 |
| | Non-Covid Natural Deaths | 28 | 3 | 1 | 10 | 2 | 3 | 0 | 0 | 13 | 1 | 4 | 0 | 0 |
| | CEMP | 10.7% | 0.0% | 0.0% | 80.0% | 0.0% | 0.0% | NA | NA | 15.4% | 0.0% | 25.0% | NA | NA |
| | RMR to unvax | | 0.0% | 0.0% | | 0.0% | 0.0% | NA | NA | | 0.0% | 162.5% | NA | NA |
| | Pfizer RMR to Moderna | | | NA | | | NA | | NA | | | NA | | NA |
| 40-59 | Covid Deaths | 15 | 0 | 1 | 27 | 0 | 1 | 0 | 0 | 13 | 1 | 1 | 0 | 0 |
| | Non-Covid Natural Deaths | 113 | 13 | 26 | 47 | 16 | 28 | 1 | 1 | 55 | 19 | 29 | 18 | 12 |
| | CEMP | 13.3% | 0.0% | 3.8% | 57.4% | 0.0% | 3.6% | 0.0% | 0.0% | 23.6% | 5.3% | 3.4% | 0.0% | 0.0% |
| | RMR to unvax | | 0.0% | 29.0% | | 0.0% | 6.2% | 0.0% | 0.0% | | 22.3% | 14.6% | 0.0% | 0.0% |
| | Pfizer RMR to Moderna | | | NA | | | NA | | NA | | | 65.5% | | NA |
| 60-79 | Covid Deaths | 40 | 1 | 6 | 51 | 8 | 7 | 1 | 0 | 45 | 3 | 5 | 2 | 4 |
| | Non-Covid Natural Deaths | 282 | 150 | 128 | 125 | 104 | 78 | 9 | 14 | 170 | 107 | 86 | 112 | 104 |
| | CEMP | 14.2% | 0.7% | 4.7% | 40.8% | 7.7% | 9.0% | 11.1% | 0.0% | 26.5% | 2.8% | 5.8% | 1.8% | 3.8% |
| | RMR to unvax | | 4.7% | 33.0% | | 18.9% | 22.0% | 27.2% | 0.0% | | 10.6% | 22.0% | 6.7% | 14.5% |
| | Pfizer RMR to Moderna | | | 703.1% | | | 116.7% | | 0.0% | | | 207.4% | | 215.4% |
| 80+ | Covid Deaths | 15 | 5 | 2 | 17 | 10 | 10 | 0 | 1 | 24 | 4 | 7 | 4 | 2 |
| | Non-Covid Natural Deaths | 279 | 354 | 129 | 141 | 210 | 66 | 21 | 18 | 180 | 113 | 78 | 316 | 127 |
| | CEMP | 5.4% | 1.4% | 1.6% | 12.1% | 4.8% | 15.2% | 0.0% | 5.6% | 13.3% | 3.5% | 9.0% | 1.3% | 1.6% |
| | RMR to unvax | | 26.3% | 28.8% | | 39.5% | 125.7% | 0.0% | 46.1% | | 26.5% | 67.3% | 9.5% | 11.8% |
| | Pfizer RMR to Moderna | | | 109.8% | | | 318.2% | | NA | | | 253.5% | | 124.4% |
| 18-59 | Covid Deaths | 18 | 0 | 1 | 35 | 0 | 1 | 0 | 0 | 15 | 1 | 2 | 0 | 0 |
| | Non-Covid Natural Deaths | 141 | 16 | 27 | 57 | 18 | 31 | 1 | 1 | 68 | 20 | 33 | 18 | 12 |
| | CEMP | 12.8% | 0.0% | 3.7% | 61.4% | 0.0% | 3.2% | 0.0% | 0.0% | 22.1% | 5.0% | 6.1% | 0.0% | 0.0% |
| | RMR to unvax | | 0.0% | 29.0% | | 0.0% | 5.3% | 0.0% | 0.0% | | 22.7% | 27.5% | 0.0% | 0.0% |
| | Pfizer RMR to Moderna | | | NA | | | NA | | NA | | | 121.2% | | NA |
| 60+ | Covid Deaths | 55 | 6 | 8 | 68 | 18 | 17 | 1 | 1 | 69 | 7 | 12 | 6 | 6 |
| | Non-Covid Natural Deaths | 561 | 504 | 257 | 266 | 314 | 144 | 30 | 32 | 350 | 220 | 164 | 428 | 231 |
| | CEMP | 9.8% | 1.2% | 3.1% | 25.6% | 5.7% | 11.8% | 3.3% | 3.1% | 19.7% | 3.2% | 7.3% | 1.4% | 2.6% |
| | RMR to unvax | | 12.1% | 31.8% | | 22.4% | 46.2% | 13.0% | 12.2% | | 16.1% | 37.1% | 7.1% | 13.2% |
| | Pfizer RMR to Moderna | | | 261.5% | | | 205.9% | | 93.8% | | | 230.0% | | 185.3% |

## Table S8. CEMP and RMR by Non-Hispanic White vs. Other Race/Ethnicity

Table and sample are similar to text Table 2, but we show results separately for non-Hispanic Whites (**Panel A**) and all other race/ethnicities (**Panel B**). Number of decedents was too small to allow for a further breakdown by race/ethnicity within Panel B.

### Panel A. Non-Hispanic White

| | | Apr-Sep 2021 | | | Oct-Dec 2021 | | | | | Jan-Jun 2022 | | | | |
|---|---|---|---|---|---|---|---|---|---|---|---|---|---|---|
| Age Bracket | Death | Unvax | M2 | P2 | Unvax | M2 | P2 | M3 | P3 | Unvax | M2 | P2 | M3 | P3 |
| 18-39 | Covid Deaths | 2 | 0 | 0 | 2 | 0 | 0 | 0 | 0 | 2 | 0 | 0 | 0 | 0 |
| | Non-Covid Natural Deaths | 22 | 3 | 1 | 2 | 4 | 2 | 0 | 0 | 10 | 3 | 2 | 0 | 1 |
| | CEMP | 9.1% | 0.0% | 0.0% | 100.0% | 0.0% | 0.0% | NA | NA | 20.0% | 0.0% | 0.0% | NA | 0.0% |
| | RMR to unvax | | 0.0% | 0.0% | | 0.0% | 0.0% | NA | NA | | 0.0% | 0.0% | NA | 0.0% |
| | Pfizer RMR to Moderna | | | NA | | | NA | | NA | | | NA | | NA |
| 40-59 | Covid Deaths | 11 | 0 | 1 | 21 | 0 | 1 | 0 | 0 | 8 | 2 | 1 | 0 | 0 |
| | Non-Covid Natural Deaths | 92 | 15 | 29 | 44 | 13 | 31 | 1 | 1 | 67 | 24 | 27 | 18 | 23 |
| | CEMP | 12.0% | 0.0% | 3.4% | 47.7% | 0.0% | 3.2% | 0.0% | 0.0% | 11.9% | 8.3% | 3.7% | 0.0% | 0.0% |
| | RMR to unvax | | 0.0% | 28.8% | | 0.0% | 6.8% | 0.0% | 0.0% | | 69.8% | 31.0% | 0.0% | 0.0% |
| | Pfizer RMR to Moderna | | | NA | | | NA | | NA | | | 44.4% | | NA |
| 60-79 | Covid Deaths | 32 | 2 | 3 | 44 | 9 | 10 | 0 | 0 | 50 | 2 | 9 | 1 | 4 |
| | Non-Covid Natural Deaths | 379 | 211 | 185 | 144 | 132 | 116 | 11 | 21 | 242 | 136 | 104 | 173 | 161 |
| | CEMP | 8.4% | 0.9% | 1.6% | 30.6% | 6.8% | 8.6% | 0.0% | 0.0% | 20.7% | 1.5% | 8.7% | 0.6% | 2.5% |
| | RMR to unvax | | 11.2% | 19.2% | | 22.3% | 28.2% | 0.0% | 0.0% | | 7.1% | 41.9% | 2.8% | 12.0% |
| | Pfizer RMR to Moderna | | | 171.1% | | | 126.4% | | NA | | | 588.5% | | 429.8% |
| 80+ | Covid Deaths | 20 | 7 | 6 | 35 | 13 | 15 | 1 | 0 | 31 | 8 | 10 | 8 | 7 |
| | Non-Covid Natural Deaths | 333 | 477 | 168 | 149 | 274 | 99 | 34 | 21 | 211 | 155 | 71 | 401 | 205 |
| | CEMP | 6.0% | 1.5% | 3.6% | 23.5% | 4.7% | 15.2% | 2.9% | 0.0% | 14.7% | 5.2% | 14.1% | 2.0% | 3.4% |
| | RMR to unvax | | 24.4% | 59.5% | | 20.2% | 64.5% | 12.5% | 0.0% | | 35.1% | 95.9% | 13.6% | 23.2% |
| | Pfizer RMR to Moderna | | | 243.4% | | | 319.3% | | 0.0% | | | 272.9% | | 171.2% |
| 18-59 | Covid Deaths | 13 | 0 | 1 | 23 | 0 | 1 | 0 | 0 | 10 | 2 | 1 | 0 | 0 |
| | Non-Covid Natural Deaths | 114 | 18 | 30 | 46 | 17 | 33 | 1 | 1 | 77 | 27 | 29 | 18 | 24 |
| | CEMP | 11.4% | 0.0% | 3.3% | 50.0% | 0.0% | 3.0% | 0.0% | 0.0% | 13.0% | 7.4% | 3.4% | 0.0% | 0.0% |
| | RMR to unvax | | 0.0% | 29.2% | | 0.0% | 6.1% | 0.0% | 0.0% | | 57.0% | 26.6% | 0.0% | 0.0% |
| | Pfizer RMR to Moderna | | | NA | | | NA | | NA | | | 46.6% | | NA |
| 60+ | Covid Deaths | 52 | 9 | 9 | 79 | 22 | 25 | 1 | 0 | 81 | 10 | 19 | 9 | 11 |
| | Non-Covid Natural Deaths | 712 | 688 | 353 | 293 | 406 | 215 | 45 | 42 | 453 | 291 | 175 | 574 | 366 |
| | CEMP | 7.3% | 1.3% | 2.5% | 27.0% | 5.4% | 11.6% | 2.2% | 0.0% | 17.9% | 3.4% | 10.9% | 1.6% | 3.0% |
| | RMR to unvax | | 17.9% | 34.9% | | 20.1% | 43.1% | 8.2% | 0.0% | | 19.2% | 60.7% | 8.8% | 16.8% |
| | Pfizer RMR to Moderna | | | 194.9% | | | 214.6% | | 0.0% | | | 315.9% | | 191.7% |

**Panel B. Other Race/Ethnicities (Black, Hispanic, and Other)**

| | | Apr-Sep 2021 | | | Oct-Dec 2021 | | | | | Jan-Jun 2022 | | | | |
|---|---|---|---|---|---|---|---|---|---|---|---|---|---|---|
| Age Bracket | Death | Unvax | M2 | P2 | Unvax | M2 | P2 | M3 | P3 | Unvax | M2 | P2 | M3 | P3 |
| 18-39 | Covid Deaths | 7 | 0 | 0 | 14 | 0 | 0 | 0 | 0 | 2 | 0 | 1 | 0 | 0 |
| | Non-Covid Natural Deaths | 41 | 2 | 4 | 24 | 1 | 4 | 0 | 0 | 22 | 1 | 11 | 1 | 1 |
| | CEMP | 17.1% | 0.0% | 0.0% | 58.3% | 0.0% | 0.0% | NA | NA | 9.1% | 0.0% | 9.1% | 0.0% | 0.0% |
| | RMR to unvax | | 0.0% | 0.0% | | 0.0% | 0.0% | NA | NA | | 0.0% | 100.0% | 0.0% | 0.0% |
| | Pfizer RMR to Moderna | | | NA | | | NA | | NA | | | NA | | NA |
| 40-59 | Covid Deaths | 27 | 0 | 0 | 36 | 1 | 0 | 0 | 0 | 15 | 1 | 2 | 0 | 0 |
| | Non-Covid Natural Deaths | 168 | 13 | 23 | 58 | 18 | 32 | 0 | 1 | 110 | 19 | 31 | 18 | 18 |
| | CEMP | 16.1% | 0.0% | 0.0% | 62.1% | 5.6% | 0.0% | NA | 0.0% | 13.6% | 5.3% | 6.5% | 0.0% | 0.0% |
| | RMR to unvax | | 0.0% | 0.0% | | 9.0% | 0.0% | NA | 0.0% | | 38.6% | 47.3% | 0.0% | 0.0% |
| | Pfizer RMR to Moderna | | | NA | | | 0.0% | | NA | | | 122.6% | | NA |
| 60-79 | Covid Deaths | 43 | 2 | 6 | 51 | 4 | 7 | 1 | 0 | 40 | 5 | 9 | 2 | 2 |
| | Non-Covid Natural Deaths | 285 | 83 | 84 | 122 | 70 | 59 | 4 | 7 | 174 | 83 | 81 | 61 | 80 |
| | CEMP | 15.1% | 2.4% | 7.1% | 41.8% | 5.7% | 11.9% | 25.0% | 0.0% | 23.0% | 6.0% | 11.1% | 3.3% | 2.5% |
| | RMR to unvax | | 16.0% | 47.3% | | 13.7% | 28.4% | 59.8% | 0.0% | | 26.2% | 48.3% | 14.3% | 10.9% |
| | Pfizer RMR to Moderna | | | 296.4% | | | 207.6% | | 0.0% | | | 184.4% | | 76.3% |
| 80+ | Covid Deaths | 12 | 1 | 1 | 14 | 2 | 7 | 0 | 1 | 25 | 3 | 6 | 1 | 0 |
| | Non-Covid Natural Deaths | 129 | 59 | 46 | 77 | 45 | 28 | 2 | 5 | 96 | 34 | 47 | 45 | 46 |
| | CEMP | 9.3% | 1.7% | 2.2% | 18.2% | 4.4% | 25.0% | 0.0% | 20.0% | 26.0% | 8.8% | 12.8% | 2.2% | 0.0% |
| | RMR to unvax | | 18.2% | 23.4% | | 24.4% | 137.5% | 0.0% | 110.0% | | 33.9% | 49.0% | 8.5% | 0.0% |
| | Pfizer RMR to Moderna | | | 128.3% | | | 562.5% | | NA | | | 144.7% | | 0.0% |
| 18-59 | Covid Deaths | 34 | 0 | 0 | 50 | 1 | 0 | 0 | 0 | 17 | 1 | 3 | 0 | 0 |
| | Non-Covid Natural Deaths | 209 | 15 | 27 | 82 | 19 | 36 | 0 | 1 | 132 | 20 | 42 | 19 | 19 |
| | CEMP | 16.3% | 0.0% | 0.0% | 61.0% | 5.3% | 0.0% | NA | 0.0% | 12.9% | 5.0% | 7.1% | 0.0% | 0.0% |
| | RMR to unvax | | 0.0% | 0.0% | | 8.6% | 0.0% | NA | 0.0% | | 38.8% | 55.5% | 0.0% | 0.0% |
| | Pfizer RMR to Moderna | | | NA | | | 0.0% | | NA | | | 142.9% | | NA |
| 60+ | Covid Deaths | 55 | 3 | 7 | 65 | 6 | 14 | 1 | 1 | 65 | 8 | 15 | 3 | 2 |
| | Non-Covid Natural Deaths | 414 | 142 | 130 | 199 | 115 | 87 | 6 | 12 | 270 | 117 | 128 | 106 | 126 |
| | CEMP | 13.3% | 2.1% | 5.4% | 32.7% | 5.2% | 16.1% | 16.7% | 8.3% | 24.1% | 6.8% | 11.7% | 2.8% | 1.6% |
| | RMR to unvax | | 15.9% | 40.5% | | 16.0% | 49.3% | 51.0% | 25.5% | | 28.4% | 48.7% | 11.8% | 6.6% |
| | Pfizer RMR to Moderna | | | 254.9% | | | 308.4% | | 50.0% | | | 171.4% | | 56.1% |

## Table S9. Non-Covid Natural Mortality Rate (Non-Covid-NMR) by Vaccine Type, Age Group, and Time Period

Table shows Non-COVID Natural Mortality Rate (NCNMR) and relative NCNMR versus the unvaccinated, for people vaccinated with 2 or 3 doses of Pfizer (P) or Moderna (M). NCNMR is defined as Non-COVID-19 natural deaths occurring among persons within indicated age group and vaccination status over the indicated period, divided by the estimated population in the same age group, vaccination status, and time period. The bottom two sets of rows aggregate results from the upper rows across broader age groups. For NCNMR ratios for the broader 18-59 and 60+ groups, *, **, *** indicate p < .05, .01, and .001, respectively; significant results (at p < .05 or better) in **boldface**. Statistical significance is assessed as follows. For each time period and broad age group, we assign the sample population to a vaccine category (Unvaccinated, Moderna 2, Pfizer 2, Moderna 3, or Pfizer 3) based on the average number of people in each category (measured at the beginning of each period). For each of these populations, we measure the number of Non-CoVID-19 natural deaths. This provides six or ten groups: three (five) groups of decedents (by vaccination status) and three (five) groups of non-decedents. We then run logistic regressions (one for each period and age group) with an outcome variable equal to 1 for the decedent groups and 0 for the remaining groups. The regressions use as predictors indicators for vaccination status (Moderna 2, Pfizer 2, Moderna 3, and Pfizer 3), with unvaccinated as the omitted category. The regressions use frequency weights, which equal the population of each group.

| Age | | Apr-Sep 2021 | | | Oct-Dec 2021 | | | | | Jan-Jun 2022 | | | | | Full Period: Apr 2021-Jun 2022 | | | | |
|---|---|---|---|---|---|---|---|---|---|---|---|---|---|---|---|---|---|---|---|
| Bracket | Measure | Unvax | M2 | P2 | Unvax | M2 | P2 | M3 | P3 | Unvax | M2 | P2 | M3 | P3 | Unvax | M2 | P2 | M3 | P3 |
| 18-39 | NCNMR | 0.04% | 0.01% | 0.01% | 0.02% | 0.01% | 0.01% | 0.00% | 0.00% | 0.03% | 0.01% | 0.02% | 0.00% | 0.00% | 0.09% | 0.03% | 0.03% | 0.00% | 0.00% |
| | NCNMR ratio to unvax | | 29.3% | 15.5% | | 49.9% | 30.5% | 0.0% | 0.0% | | 41.4% | 60.7% | 15.5% | 15.3% | | 38.3% | 34.5% | 5.3% | 5.3% |
| | NCNMR ratio to Mod | | | 52.8% | | | 61.2% | | NA | | | 146.7% | | 98.9% | | | 90.3% | | 98.9% |
| 40-59 | NCNMR | 0.27% | 0.09% | 0.08% | 0.15% | 0.07% | 0.09% | 0.01% | 0.01% | 0.31% | 0.19% | 0.13% | 0.14% | 0.09% | 0.74% | 0.34% | 0.29% | 0.15% | 0.10% |
| | NCNMR ratio to unvax | | 31.4% | 28.1% | | 45.3% | 55.5% | 6.3% | 5.9% | | 60.0% | 42.2% | 44.1% | 28.1% | | 46.5% | 39.8% | 20.0% | 13.1% |
| | NCNMR ratio to Mod | | | 89.4% | | | 122.5% | | 94.4% | | | 70.3% | | 63.6% | | | 85.7% | | 65.6% |
| 60-79 | NCNMR | 1.78% | 0.66% | 0.44% | 0.98% | 0.56% | 0.40% | 0.09% | 0.10% | 1.69% | 1.69% | 0.98% | 0.66% | 0.47% | 4.46% | 2.90% | 1.82% | 0.75% | 0.57% |
| | NCNMR ratio to unvax | | 36.9% | 24.9% | | 56.5% | 40.2% | 8.6% | 9.8% | | 99.6% | 57.9% | 39.0% | 28.0% | | 65.1% | 40.8% | 16.7% | 12.8% |
| | NCNMR ratio to Mod | | | 67.5% | | | 71.1% | | 113.8% | | | 58.1% | | 71.8% | | | 62.7% | | 76.6% |
| 80+ | NCNMR | 4.57% | 4.41% | 2.04% | 2.55% | 3.34% | 1.88% | 0.72% | 0.45% | 3.85% | 5.32% | 4.07% | 5.04% | 2.97% | 10.97% | 13.08% | 7.99% | 5.76% | 3.42% |
| | NCNMR ratio to unvax | | 96.5% | 44.7% | | 131.1% | 73.7% | 28.2% | 17.7% | | 138.3% | 105.7% | 131.1% | 77.1% | | 119.2% | 72.9% | 52.6% | 31.2% |
| | NCNMR ratio to Mod | | | 46.4% | | | 56.2% | | 62.6% | | | 76.4% | | 58.8% | | | 61.1% | | 59.3% |
| 18-59 | NCNMR | 0.12% | 0.07% | 0.05% | 0.07% | 0.04% | 0.04% | 0.01% | 0.01% | 0.13% | 0.09% | 0.06% | 0.10% | 0.05% | 0.32% | 0.19% | 0.15% | 0.11% | 0.06% |
| | NCNMR ratio to unvax | | **57.1%*** | **37.7%***** | | **58.2%***** | **61.0%***** | **14.5%***** | **7.6%***** | | **66.4%**** | **48.1%***** | **80.2%**** | **42.0%***** | | **61.1%***** | **46.8%***** | **35.7%***** | **18.7%*** |
| | NCNMR ratio to Mod | | | **65.9%***** | | | 104.6% | | 52.2% | | | 72.5% | | 52.4% | | | **76.6%***** | | **52.4%***** |
| 60+ | NCNMR | 2.36% | 1.47% | 0.68% | 1.37% | 1.12% | 0.59% | 0.21% | 0.15% | 2.22% | 2.45% | 1.38% | 1.54% | 0.83% | 5.95% | 5.04% | 2.65% | 1.75% | 0.98% |
| | NCNMR ratio to unvax | | **62.2%***** | **28.7%***** | | **81.6%**** | **43.0%***** | **15.5%***** | **11.1%***** | | 110.3% | **62.1%***** | **69.2%***** | **37.3%***** | | **84.6%***** | **44.5%***** | **29.4%***** | **16.5%***** |
| | NCNMR ratio to Mod | | | **46.2%***** | | | **52.7%***** | | **71.3%***** | | | **56.3%***** | | **53.9%***** | | | **52.6%***** | | **56.0%***** |

# Table S10. Covid-19 PFR by Age Group and Time Period

Sample is same as text Table 2 and format is similar, but with a different outcome variable: COVID-19 Population Fatality Rate (Covid-PFR) instead of CEMP.

| Age | | Apr-Sep 2021 | | | Oct-Dec 2021 | | | | | Jan-Jun 2022 | | | | |
|---|---|---|---|---|---|---|---|---|---|---|---|---|---|---|
| Bracket | Measure | Unvax | M2 | P2 | Unvax | M2 | P2 | M3 | P3 | Unvax | M2 | P2 | M3 | P3 |
| 18-39 | Covid PFR | 0.006% | 0.000% | 0.000% | 0.013% | 0.000% | 0.000% | 0.000% | 0.000% | 0.004% | 0.000% | 0.001% | 0.000% | 0.000% |
| | RMR to unvax | | 0.0% | 0.0% | | 0.0% | 0.0% | 0.0% | 0.0% | | 0.0% | 35.1% | 0.0% | 0.0% |
| | Pfizer RMR to Moderna | | | NA | | | NA | | NA | | | NA | | NA |
| 40-59 | Covid PFR | 0.046% | 0.000% | 0.001% | 0.087% | 0.002% | 0.002% | 0.000% | 0.000% | 0.039% | 0.011% | 0.006% | 0.000% | 0.000% |
| | RMR to unvax | | 0.0% | 2.8% | | 2.4% | 1.7% | 0.0% | 0.0% | | 26.8% | 14.7% | 0.0% | 0.0% |
| | Pfizer RMR to Moderna | | | NA | | | 71.4% | | NA | | | 54.8% | | NA |
| 60-79 | Covid PFR | 0.217% | 0.008% | 0.014% | 0.354% | 0.034% | 0.039% | 0.005% | 0.000% | 0.364% | 0.043% | 0.083% | 0.009% | 0.013% |
| | RMR to unvax | | 3.8% | 6.3% | | 9.5% | 11.1% | 1.3% | 0.0% | | 11.8% | 22.8% | 2.4% | 3.5% |
| | Pfizer RMR to Moderna | | | 167.8% | | | 117.2% | | 0.0% | | | 192.3% | | 145.4% |
| 80+ | Covid PFR | 0.329% | 0.063% | 0.065% | 0.556% | 0.167% | 0.336% | 0.017% | 0.016% | 0.690% | 0.283% | 0.496% | 0.103% | 0.087% |
| | RMR to unvax | | 19.3% | 19.8% | | 30.1% | 60.3% | 3.1% | 2.8% | | 41.0% | 71.9% | 15.0% | 12.6% |
| | Pfizer RMR to Moderna | | | 102.6% | | | 200.8% | | 91.3% | | | 175.6% | | 84.0% |
| 18-59 | Covid PFR | 0.020% | 0.000% | 0.001% | 0.039% | 0.001% | 0.001% | 0.000% | 0.000% | 0.016% | 0.005% | 0.003% | 0.000% | 0.000% |
| | RMR to unvax | | 0.0% | 2.9% | | 2.7% | 1.6% | 0.0% | 0.0% | | 28.9% | 19.3% | 0.0% | 0.0% |
| | Pfizer RMR to Moderna | | | NA | | | 61.5% | | NA | | | 66.7% | | NA |
| 60+ | Covid PFR | 0.242% | 0.020% | 0.021% | 0.40% | 0.06% | 0.08% | 0.01% | 0.00% | 0.44% | 0.09% | 0.14% | 0.03% | 0.02% |
| | RMR to unvax | | 8.1% | 8.7% | | 14.9% | 19.2% | 1.8% | 0.6% | | 20.7% | 30.5% | 6.3% | 5.3% |
| | Pfizer RMR to Moderna | | | 108.1% | | | 128.8% | | 33.3% | | | 147.6% | | 83.7% |

## Figure S1. Sample selection flowchart

Chart shows the process for selecting the sample of decedents in Milwaukee County used in the Pfizer v. Moderna vaccine effectiveness analysis. After identifying natural deaths in each time periods, we record natural deaths by number of vaccine doses (0, 1, 2, or 3 doses) and keep only the unvaccinated and people who received 2 or more doses of only Pfizer or only Moderna. Text Table 2 reports the number of Covid-19 deaths and other natural deaths by period, age bin, and vaccination status.

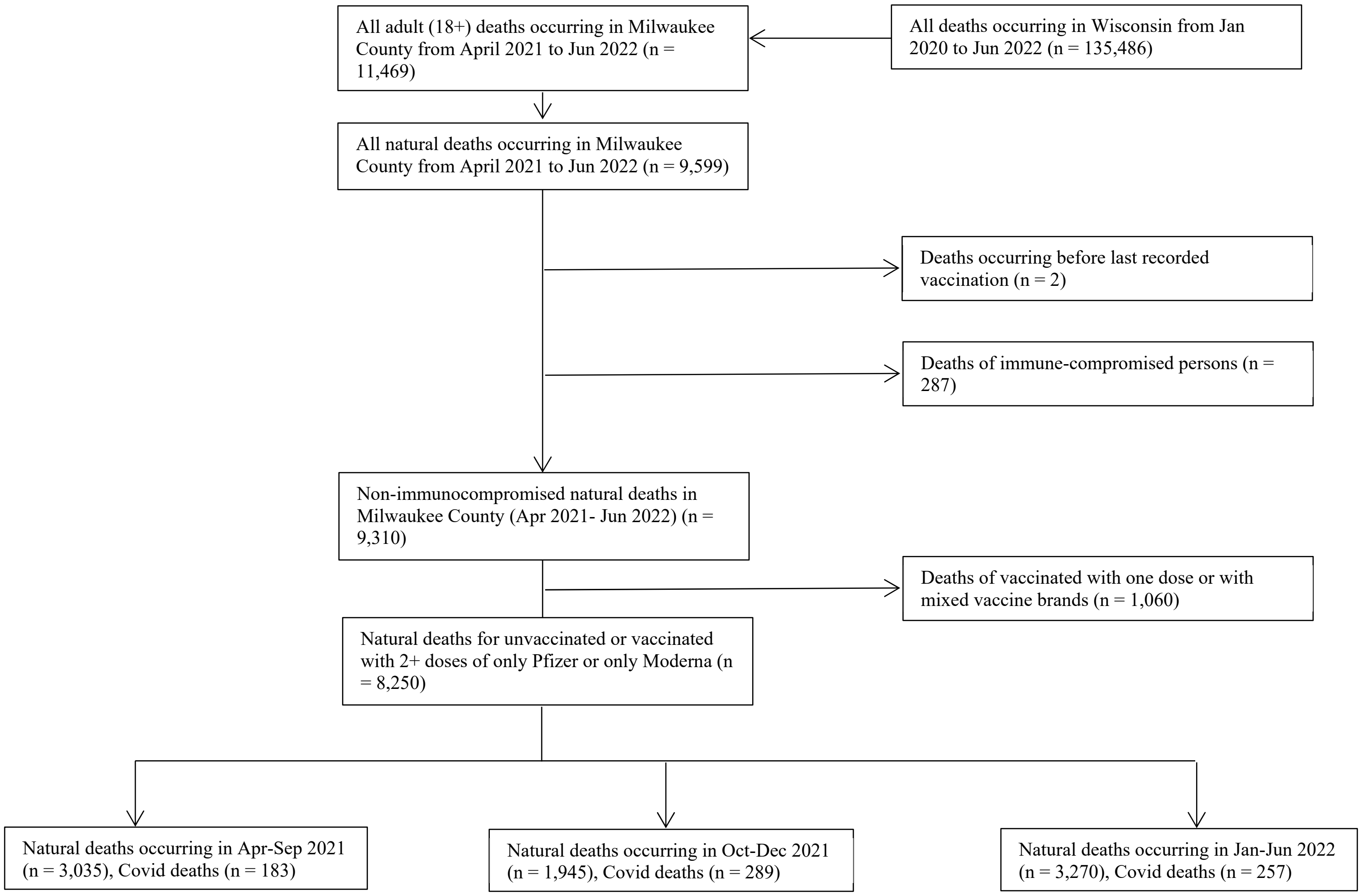

### Figure S2. Actual versus Predicted Non-COVID Natural Mortality in Wisconsin

**Panel A** shows monthly data for natural non-COVID-19 and all natural deaths, for Wisconsin for January 2017 – June 2022. For the pandemic period starting March 2020, figure shows both actual and predicted natural non-COVID deaths. Predicted deaths are based on linear extrapolation from 2017-2019 to the same calendar month during the pandemic period. Natural deaths (including COVID-19 deaths) are shown as solid red line, which shows COVID-related peaks in late 2020 and late-2021-early 2022. Natural non-COVID-19 deaths (all natural deaths minus COVID-19 deaths) are shown as solid blue line. Predicted natural non-COVID deaths are shown as dashed green line. **Panel B** is similar but is limited to Milwaukee County over 2020-June 320, 20220, omits total natural deaths, and adds 95% confidence interval for predicted non-COVID natural deaths.

**Panel A. Wisconsin 2017-June 2022**

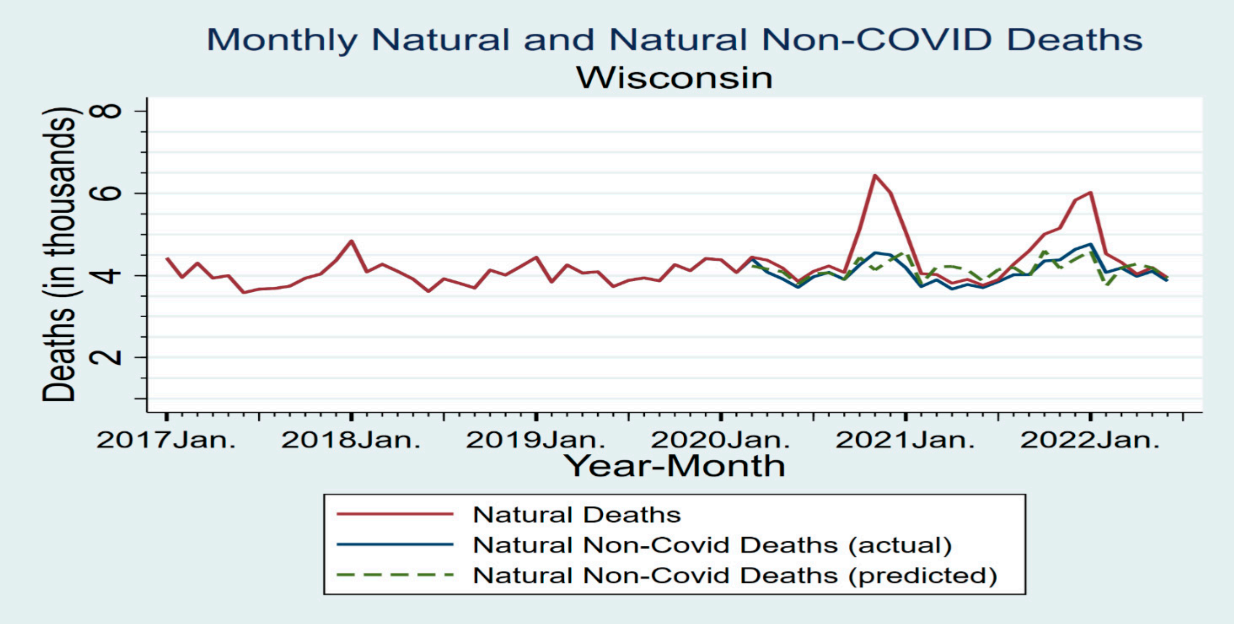


**Panel B. Milwaukee County, 2020-June 2022**

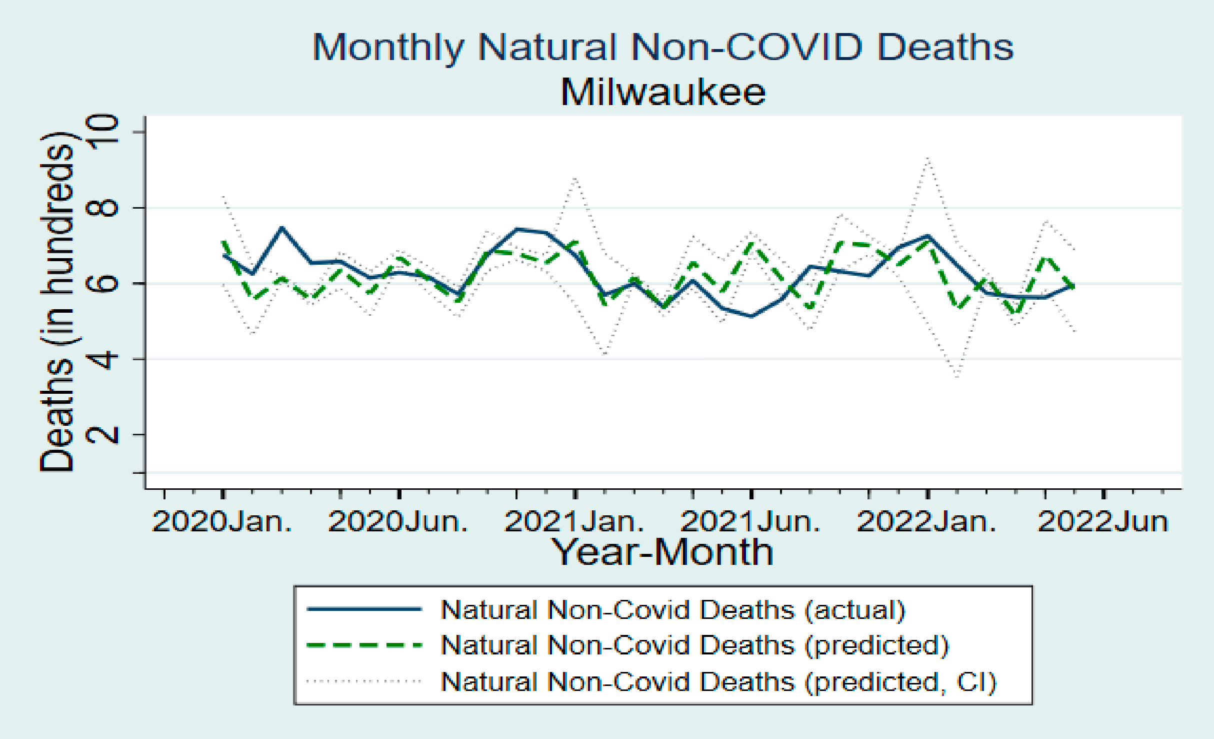

**Figure S3. Actual versus Predicted Non-COVID-19 Natural Mortality Rates: Indiana**

Figure is similar to Figure App-2 (for Wisconsin) excepts shows out-of-sample analysis for Indiana (for which we have similar data). All natural deaths are shown as solid red line; natural non-COVID-19 as solid blue line and predicted natural non-COVID deaths as dashed green line.

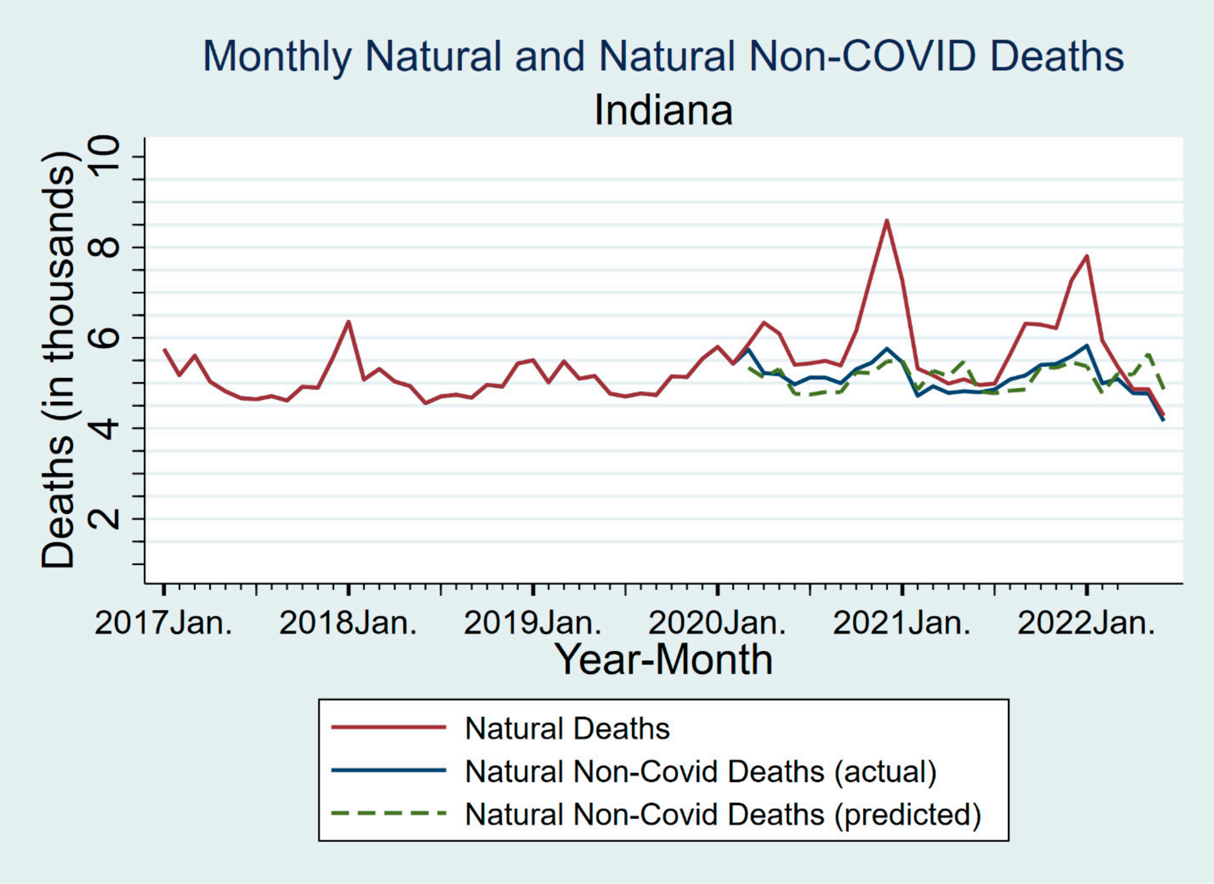

## Figure S4.  Vaccination Rates for Adults by Age Group in Milwaukee County

Vaccination percentages over time for Milwaukee County residents, through June 30, 2022.

### Panel A.  Full-vaccination rates

Figure shows full vaccination rates over time in Milwaukee by age range.  Full vaccination is defined as 1 J&J dose, 2 mRNA doses (Pfizer, Moderna, or mixed), or more.

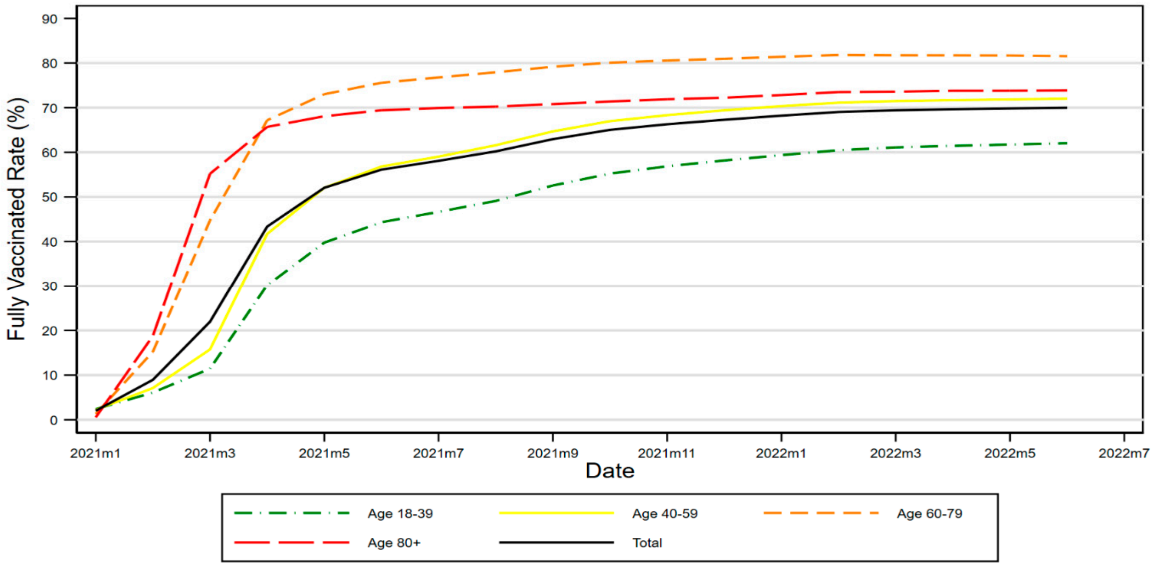


### Panel B.  Three Dose Vaccination Rates

Figure shows three-dose vaccination rates over time in Milwaukee by age range, as percentage of people receiving two vaccine doses.

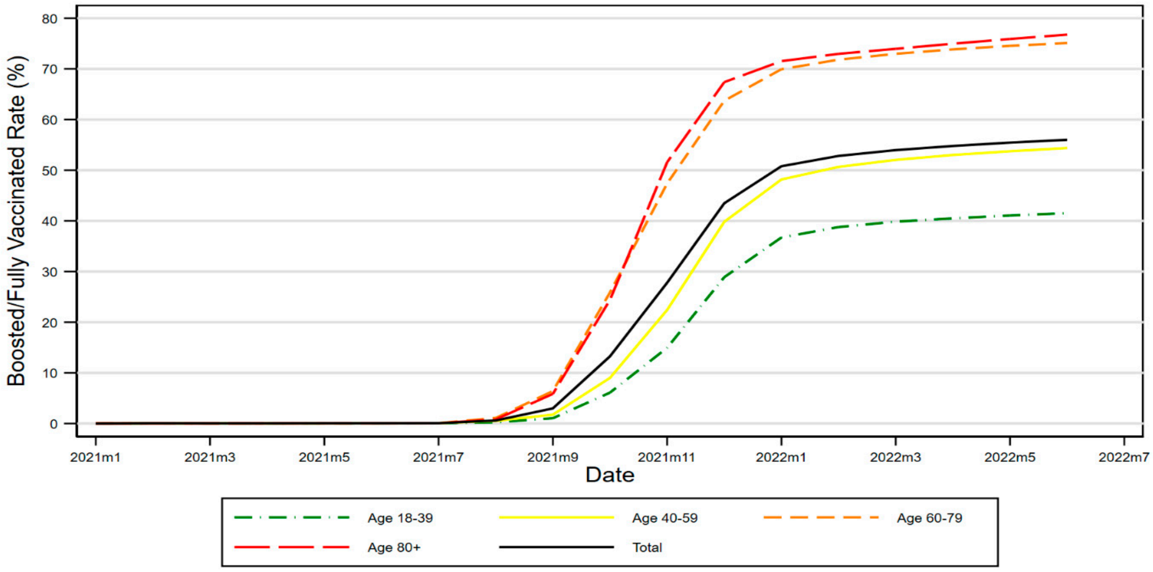

**Figure S5. Correlation between 2019 Natural Mortality and 2020 COVID-19 Mortality**

**Top panel**: Scatterplot of natural mortality in Milwaukee County over April-December 2019 against COVID-19 mortality over April-December 2020, for groups defined by age (18-39, 40-49, 50-59, 60-69, 70-79, 80-89, 90+)*gender*race/ethnicity, best-fit regression line, and Pearson correlation coefficient. **Bottom panel:** Similar but shows the relation between Covid Deaths in 2020 and All Natural Deaths in 2019, and adds colored points for Blacks (orange squares) and Hispanics (blue triangles).

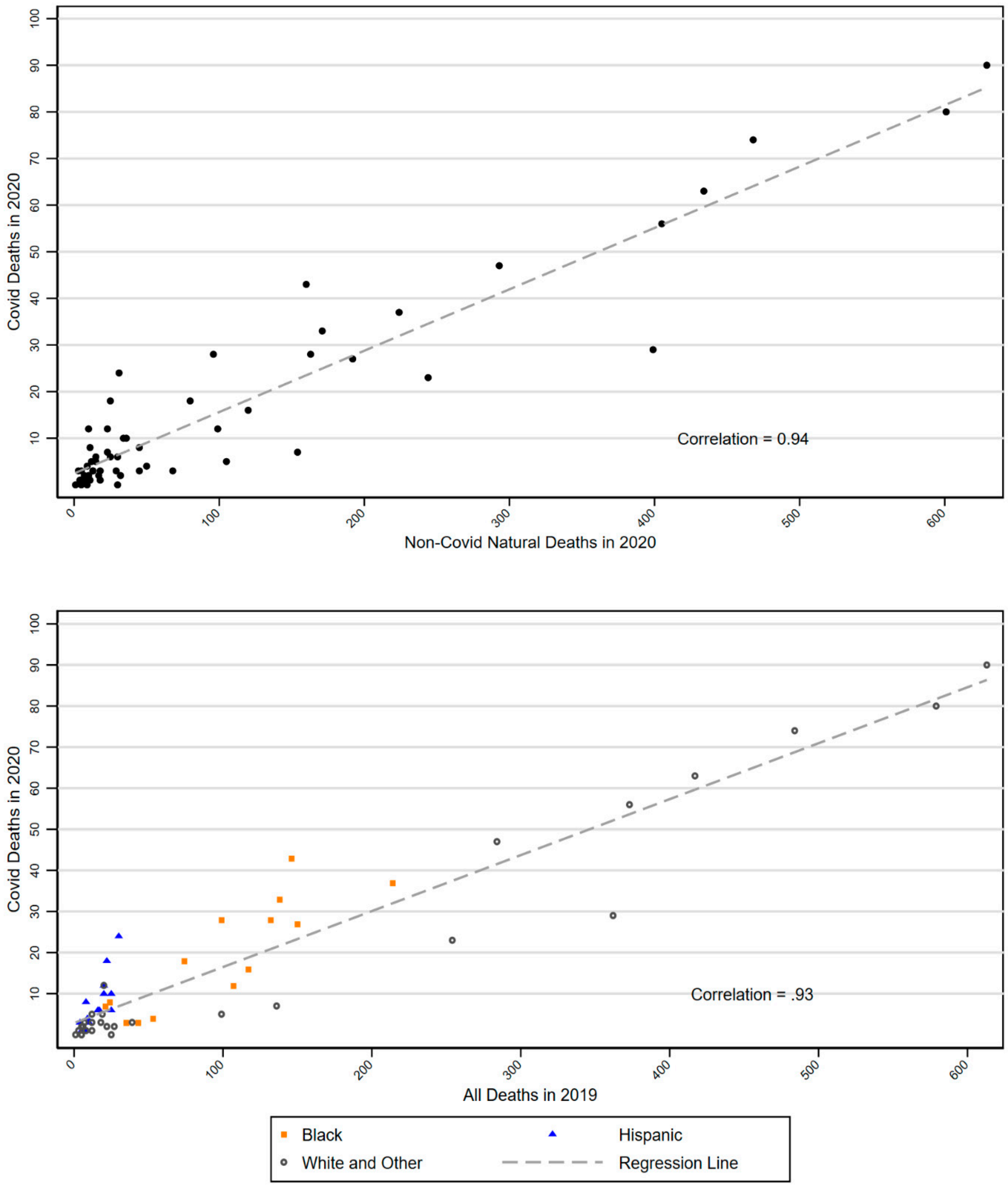

Supplement: Supplementary file 1 [file vaccines-11-00971-s001.zip › vaccines-2338972-supplementary.pdf]
